# Fraction of cancer incidence and mortality attributable to dietary factors in Korea from 2015 to 2030

**DOI:** 10.4178/epih.e2025065

**Published:** 2025-12-08

**Authors:** Hyun Jeong Cho, Jin Young Yoo, Ga-Eun Yie, An Na Kim, Soseul Sung, Sungji Moon, Youjin Hong, Sangjun Lee, Inah Kim, Kwang-Pil Ko, Sun-Seog Kweon, Jung Eun Lee, Sue K. Park

**Affiliations:** 1 Division of Prevention, National Cancer Center Institute for Cancer Control, Tokyo, Japan; 2 Department of Food and Nutrition, Seoul National University College of Human Ecology, Seoul, Korea; 3 Department of Preventive Medicine, Seoul National University College of Medicine, Seoul, Korea; 4 Cancer Research Institute, Seoul National University, Seoul, Korea; 5 Department of Biomedical Sciences, Seoul National University Graduate School, Seoul, Korea; 6 Interdisciplinary Program in Cancer Biology, Seoul National University College of Medicine, Seoul, Korea; 7 Integrated Major in Innovative Medical Science, Seoul National University Graduate School, Seoul, Korea; 8 Department of Occupational and Environmental Medicine, Hanyang University College of Medicine, Seoul, Korea; 9 Clinical Preventive Medicine Center, Seoul National University Bundang Hospital, Seongnam, Korea; 10 Department of Preventive Medicine, Chonnam National University Medical School, Hwasun, Korea; 11 Research Institute of Human Ecology, Seoul National University, Seoul, Korea

**Keywords:** Diet, Carcinoma, Public health

## Abstract

**OBJECTIVES:**

Dietary factors play an important role in modifying cancer risk. This study aimed to assess the proportion of cancer incidence and mortality in Korea attributable to dietary factors from 2015 to 2030.

**METHODS:**

We estimated the population-attributable fraction (PAF) of cancer incidence and mortality using dietary intake levels, exposure rates, and relative risks (RRs). Intake and exposure rates were derived from the Korean National Health and Nutrition Examination Survey, assuming a 15-year latency. RRs were obtained from meta-analyses of Korean cohort and case- control studies.

**RESULTS:**

In 2020, 6.08% of cancer cases and 5.70% of deaths in Korea were attributable to dietary factors. High salted vegetable intake (2.12% for incidence and 1.78% for deaths) and low intake of non-starchy vegetables and fruits (1.92 and 2.34%, respectively) were major contributors. However, high intakes of red meat and processed meat showed low PAFs, each less than 1%. The projected PAF for cancer attributable to high salted vegetable intake is expected to decrease substantially to 1.17% in 2030. In contrast, the PAF linked to low intake of non-starchy vegetables and fruits is projected to remain relatively stable.

**CONCLUSIONS:**

Our findings provide evidence that dietary factors make a substantial contribution to cancer incidence and mortality in Korea. This study highlights that reducing salted vegetable intake and encouraging a diet rich in non-starchy vegetables and fruits may support cancer prevention efforts. Continuous monitoring of dietary trends remains crucial for reducing the cancer burden.

## GRAPHICAL ABSTRACT

[Fig f4-epih-47-e2025065]

## Key Message

In 2020, dietary factors accounted for a proportion of the cancer burden, with high intake of salted vegetables and low intake of non-starchy vegetables and fruits showing the largest contributions among dietary factors examined. These findings suggest the need for continued assessment of dietary patterns and their potential relevance in public health discussions on cancer prevention.

## INTRODUCTION

Cancer is one of the leading causes of death worldwide, with 10 million cancer deaths reported in 2020 [1]. In Korea, cancer ranked as the first cause of death, with an age-standardized incidence rate of 262.2 per 100,000 in 2020 [2]. Newly diagnosed cancer cases are predicted to rise from 19.3 million in 2020 to 28.4 million in 2040 [1].

Diet plays an important role in modifying cancer risk. The World Cancer Research Fund (WCRF) report found that processed meat has been associated with a higher colorectal cancer risk, while dietary fiber and whole grains have been associated with lower risk [3]. The International Agency for Research on Cancer (IARC) classified processed meat as carcinogenic and red meat as probably carcinogenic [4]. The Global Burden of Disease (GBD) study estimated that dietary risks accounted for approximately 5% of global cancer disability-adjusted life years in 2019 [5].

Estimating the cancer burden attributable to diet is critical for effective prevention programs and public health planning. Because dietary patterns differ across countries, country-specific estimates are necessary. In Korea, salted vegetable intake remains high and dietary patterns have changed in recent decades, with intake measuring around 136.6 g/day in male and 90.4 g/day in female in 2015. Meanwhile, red and processed meat intake increased from roughly 48-76 g/day to 51-83 g/day for red meat and from 3-5 g/day to 8-13 g/day for processed meat between 2005 and 2015, based on our analysis of Korean National Health and Nutrition Examination Survey (KNHANES) data. These trends underscore the need for Korea-specific assessments. Previous studies have examined the burden of cancers attributable to dietary factors in the United States [6], the United Kingdom [7], France [8], Germany [9], Australia [10], Brazil [11], Japan [12], and China [13]. However, in Korea, although several reports have assessed other risk factors, studies estimating the preventive fraction of diet remain limited [14-20]. Most previous Korean work has focused on exposures such as excess body weight, hormone and reproductive factors, or carcinogenic drugs, and diet has not been comprehensively addressed. Therefore, we aimed to estimate the proportion of cancer incidence and mortality attributable to dietary factors among the Korean population from 2015 to 2030, with the goal of providing evidence to support cancer prevention and public health strategies.

## MATERIALS AND METHODS

### Definition of dietary factors

Dietary factors were selected based on 2 criteria: (1) evidence for causal links to cancers from the WCRF Cancer Update Program (CUP) [3] and (2) classification as Group 1 carcinogens by the IARC Monograph [4]. The selected risk factors included high intakes of red meat, processed meat, salted vegetables, and salted fish, as well as low intakes of dietary fiber and non-starchy vegetables and fruits (Supplementary Material 1).

Dietary factors such as calcium supplements (colorectal cancer), glycemic load (endometrial cancer), high-dose beta-carotene supplements (lung cancer), mate (esophageal cancer), and iodine deficiency (thyroid cancer) were excluded because representative population-level data were unavailable. Coffee (liver and endometrial cancers) and dairy products (colorectal cancer) were also excluded due to concerns about substantial discrepancies between reported and usual intake when relying on a single 24-hour recall method [21,22]. Whole grains (colorectal cancer), although an important dietary component, were excluded because their contribution was indirectly captured through dietary fiber intake. Although the WCRF CUP identified foods preserved by salting as a probable risk factor for stomach cancer, we separated them into salted vegetables and salted fish to account for differences in serving size. For the dietary factors included, population-attributable fractions (PAFs) of cancer were estimated using national intake data, Korean cohort-based relative risks (RRs), and cancer registry statistics.

### Dietary intake estimation

Dietary factors were defined as follows: salted vegetables (including kimchi and other pickled vegetables); salted fish (salt-preserved fish); red meat (pork, beef, and lamb); processed meat (ham, bacon, and sausages); and non-starchy vegetables and fruits (a broad range of fresh vegetables and fruits excluding starchy tubers such as potatoes). Dietary intake levels for adults aged 20 years and older were estimated using one-day 24-hour recall data from the KNHANES for 2001, 2005, and 2007-2018. Age-standardized intake levels were calculated using the 2000 Census population as the standard. Intake levels for 2000 were predicted using a linear regression model. For dietary fiber, KNHANES data from 2013-2018 were used because earlier data were unavailable. The prevalence of dietary risk factors was estimated using optimal intake levels from the GBD 2017 study [23] or average intake values from global studies, representing the proportion of individuals whose intake exceeded or fell below optimal levels.

### Estimation of relative risks for cancer risk

We conducted dose-response meta-analyses of Korean cohort and case-control studies to estimate RRs for cancer based on incremental changes in dietary intake. For sensitivity analyses, the meta-analyses were expanded to include Asian cohort and case-control studies, as well as global prospective cohort studies. Associations between dietary factors and cancer risk were examined for the following relationships: (1) red meat and colorectal cancer, (2) processed meat and colorectal cancer, (3) salted vegetables and stomach cancer, (4) salted fish and nasopharyngeal cancer, (5) salted fish and stomach cancer, (6) dietary fiber and colorectal cancer, and (7) non-starchy vegetables and fruits (combined) and aerodigestive and other cancers (colorectal, stomach, and lung cancers).

A literature search was performed using PubMed, Embase, and KoreaMed up to November 2019 to identify prospective cohort and Asian case-control studies. Articles were selected according to the following criteria: (1) evaluation of dietary factors and cancer risk (incidence or mortality); (2) prospective cohort or Asian case-control design; (3) written in English or Korean; (4) provision of dose-response RRs with 95% confidence intervals (CIs); and (5) inclusion of ≥1,000 cases for hospital-based studies. When multiple studies were duplicated, preference was given to analyses with more cases or more recent publication dates.

Because Korean cohort studies were limited, additional analyses were conducted using available cohort data from the Korean Cohort Consortium [24], which included the Korean Multi-Center Cancer Cohort [25], the Namwon study/Dong-gu study [26], the Korean Genome and Epidemiology Study [27], and KNHANES data from the Korea Disease Control and Prevention Agency [28]. Analyses were adjusted for potential confounders, including age, sex, cohort, survey year, body mass index, smoking, alcohol use, education, physical activity, and energy intake, depending on the availability of variables in each study.

We estimated sex-combined and sex-specific RRs using a random-effects model for cancer incidence and mortality. Heterogeneity across studies was tested using the Cochran Q-test and the *I*^2^ statistic [29]. Studies with high heterogeneity (p<0.05 or *I*^2^>50%) were excluded from the meta-analyses. All meta-analyses were performed using R version 4.0.2 (R Foundation for Statistical Computing, Vienna, Austria).

### Calculation of cancer population-attributable fractions (PAF) and prediction of PAF values in 2025 and 2030

We calculated PAF of specific cancers attributed to dietary factors using the following modified Levin’s formula (equation 1) [30].


(1) $$PAF=\frac{P\left(e^{\beta *\text{dose}}-1\right)}{P\left(e^{\beta *\text{dose}}-1\right)+1}$$

where *P* represents the proportion of individuals with intake levels outside the optimal dietary range, dose is the average intake level of each dietary factor, and β=log(RR) represents the RR per unit increment of intake. Monte Carlo simulation methods were used to estimate 95% CIs [31,32], repeating the estimation 10,000 times and using the 2.5th percentile and 97.5th percentile as the 95% CI.

The PAF calculation involved 2 steps: (1) estimating attributable cases (ACs) for specific cancers associated with each dietary factor and then summing them to obtain the total AC, and (2) dividing the total number of ACs by the total number of cancer cases or deaths to obtain the PAF. PAFs were estimated for 2015, 2020, 2025, and 2030 to assess temporal trends. Cancer incidence and mortality data for adults aged 20 years and older in 2015 and 2020 were obtained from the National Cancer Registration Statistics at the National Cancer Center and the Cause of Death Statistics from Statistics Korea [33]. PAF projections for 2025 and 2030 were based on the assumption of a 15-year latency period and stable RRs. Methods for estimating projected population counts and expected numbers of cancer cases and deaths in 2025 and 2030 have been described in previous studies [34].

### Ethics statement

This study received approval from the Institutional Review Board at Seoul National University Hospital (E-1903-024-1015).

## RESULTS

### Exposure rates

From 2000 to 2030, the prevalence of red and processed meat intake increased, while the prevalence of salted vegetables and salted fish steadily decreased. Only minimal changes were observed for dietary fiber and non-starchy vegetables and fruits (Supplementary Material 2). The prevalence rates in 2000 and 2030 were as follows (Supplementary Material 3): red meat increased in both male (52.6 to 67.4%) and female (36.7 to 51.6%); processed meat rose markedly in male (4.4 to 36.8%) and female (4.7 to 33.3%); salted vegetables decreased in male (99.0 to 83.2%) and female (95.8 to 74.5%); salted fish declined modestly in male (9.7 to 7.0%) and female (8.6 to 6.7%). Dietary fiber intake decreased in male (37.6 to 30.2%) but remained essentially unchanged in female (48.3 to 48.1%). Non-starchy vegetable and fruit intake showed slight decreases in male (79.2 to 73.5%) and female (76.7 to 74.1%).

### Relative risks of cancer

We estimated the RRs of dietary factors associated with cancer risk through meta-analyses of Korean cohort and case-control studies, supplemented by Asian or global cohort studies when needed for PAF calculation (Supplementary Material 4). Our findings showed significantly increased risks of colorectal cancer with each 50 g/day increase in processed meat intake and of nasopharyngeal cancer with each 20 g/day increase in salted fish intake. Sensitivity analyses using Asian cohort studies produced similar patterns. When using RRs from global cohort studies, stronger associations were observed for red meat and dietary fiber with colorectal cancer compared to Asian estimates. For example, colorectal cancer incidence increased by 21% per 120 g/day of red meat (RR, 1.21; 95% CI, 1.07 to 1.38) among female, and dietary fiber intake reduced incidence (RR, 0.94; 95% CI, 0.92 to 0.97) and mortality by 25% (95% CI, 5 to 41) per 10 g/day among male.

### Population-attributable fraction of cancer

In 2020, dietary factors accounted for 6.08% of cancer incidence (8.43% for male and 3.45% for female) and 5.70% of cancer mortality (7.93% for male and 2.08% for female) (Table 1, Supplementary Material 5). Dietary risk-increasing factors contributed 2.25% of incidence (3.13% for male and 1.28% for female), while protective dietary factors accounted for 3.82% (5.30% for male and 2.18% for female). For cancer mortality, the respective contributions were 2.31% (2.76% for male and 1.59% for female) and 3.39% (5.17% for male and 0.49% for female). By 2025 and 2030, PAFs for cancer incidence decreased, whereas PAFs for mortality remained largely stable.

**Figure 1.**
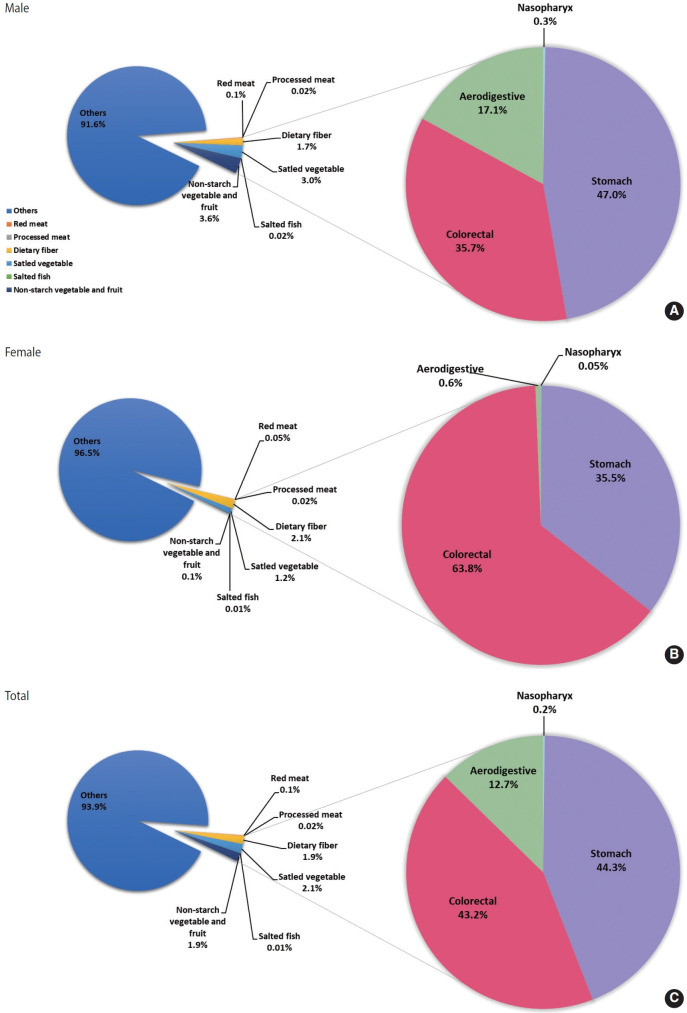
The population-attributable fraction of cancer cases attributable to dietary factors and the proportion of specific cancers among all diet-related cancer cases in Korea, 2020. (A) Male cancer cases. (B) Female cancer cases. (C) Total cancer cases.

**Figure 2.**
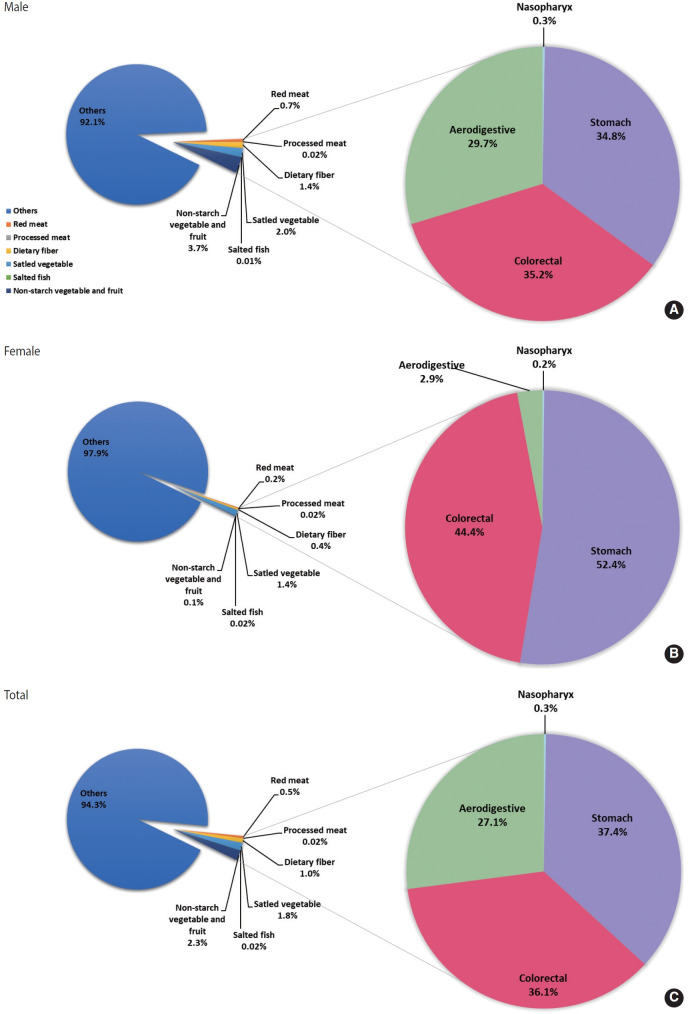
The population-attributable fraction of cancer deaths attributable to dietary factors and the proportion of specific cancers among all diet-related cancer deaths in Korea, 2020. (A) Male cancer deaths. (B) Female cancer deaths. (C) Total cancer deaths.

From 2015 to 2030, PAFs for high red meat intake increased slightly from 0.08% to 0.10%, and processed meat from 0.01% to 0.08%. PAFs for salted vegetables decreased substantially from 3.00% to 1.17%, and the PAF for low intake of non-starchy vegetables and fruits decreased from 2.22% to 1.92%. Dietary fiber PAFs peaked at 1.90% in 2020 and then declined to 1.54% in 2025 and 1.61% in 2030. For deaths, PAFs for dietary fiber increased to 1.29% in 2030, and PAFs for low non-starchy vegetables and fruits increased to 2.84% in 2030. In 2020, stomach cancer had the highest diet-related PAF, accounting for 24.61% of cancer incidence and 24.27% of cancer deaths (Table 2, Supplementary Material 6). Colorectal cancer followed, with PAFs of 18.38% for incidence and 18.57% for deaths, while nasopharyngeal cancer accounted for 6.97% and 7.43%, and aerodigestive cancers for 5.12% and 5.46%. From 2015 to 2030, PAFs for nasopharyngeal and stomach cancers declined, whereas colorectal cancer PAFs increased from 18.75% to 20.03%. The PAF for aerodigestive cancers remained stable. Among attributable cancer cases related to dietary factors, stomach cancer accounted for 44.3% of incident cases and 37.4% of deaths (Figures 1 and 2). The distribution varied modestly by sex: 47.0% for male and 35.5% for female for incident cases, and 34.8% for male and 52.4% for female for deaths.

When estimating PAFs attributable to all combined dietary factors, we conducted sensitivity analyses using Asian and global RRs (Table 3). The PAFs in 2020 were 4.99% for incident cases and 4.21% for deaths when applying Asian RRs, and 3.76% for incident cases and 5.09% for deaths when applying global RRs. These values were approximately 1-2 percentage points lower than estimates based on Korean RRs (6.08% for incidence and 5.70% for deaths), reflecting modest variation across RR sources and supporting the robustness of the findings. These small differences may partially reflect the limited number of studies contributing to the Asian or Korean RRs. Sensitivity analyses using 2000-2015 intake data produced similar patterns, with slightly higher PAFs under a 20-year latency assumption and comparable values under a 10-year latency, consistent with the long-term nature of diet–cancer associations (Supplementary Material 7).

## DISCUSSION

**Figure 3.**
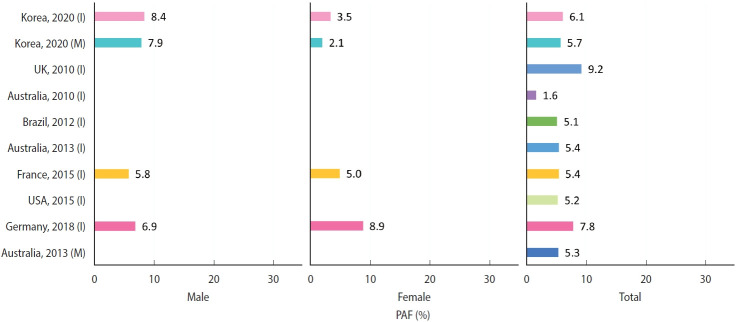
International comparison of the population-attributable fraction (PAF) of cancer cases and deaths attributable to dietary factors. (I) indicates incidence and (M) indicates mortality.

In 2020, dietary factors accounted for 6.08% of cancer incidence and 5.70% of cancer deaths in Korea, with greater contributions observed in male (8.43% for incidence and 7.93% for deaths) than in female (3.45% for incidence and 2.08% for deaths). Stomach and colorectal cancers showed the highest proportions of diet-attributable cases and deaths, driven primarily by high intake of salted vegetables and low intake of non-starchy vegetables and fruits. These results are broadly consistent with international estimates of cancer burden attributable to diet. Our findings were comparable to those reported for all diet-related cancer incidence in the United States (5.2%) [6], United Kingdom (9.2%) [7], France (5.4%) [8], Germany (7.8%) [9], Australia (5.4%) [10], and Brazil (5.1%) [11] (Figure 3).

The 2 major dietary contributors to cancer burden in Korea were high intake of salted vegetables and low intake of non-starchy vegetables and fruits, which is consistent with findings from the Eastern Mediterranean region [35]. That study identified low fruit intake as the dietary factor with the highest PAF (6.0% for male and 3.5% for female), followed by low vegetable intake (4.9% for male and 2.7% for female), high salt intake (2.0% for male and 0.9% for female), processed meat (0.2% for both sexes), and red meat (0.2% for male and 0.1% for female). Across other multi-factor dietary burden studies, the dietary factor with the highest PAF for all cancer incidence varied: whole grains in the United States (1.81%) [6], dietary calcium in Canada (Alberta) (0.9%) [36] and Brazil (0.9%) [11], red and processed meat in the United Kingdom (2.7%) [37], and dietary fiber in France (1.4%) [8] and Germany (3.3%) [9].

When comparing our estimates for salted vegetables (2.12% for incidence and 1.78% for deaths) with PAFs for salt intake in 30countries lacking estimates specifically for salted vegetables, our PAFs were higher than those reported in Japan (1.6% for incidence and 1.4% for deaths) [12], Canada (Alberta) (0.2% for incidence) [36], the United Kingdom (0.5% for incidence) [37], Germany (0.3% for incidence) [9], and the Eastern Mediterranean region (2.0% for male and 0.9% for female for incidence) [35]. The higher PAFs observed in Korea likely reflect the notably high intake of salted vegetables, which reached 145.9 g/day in 2005. In Japan, estimated intakes of pickled vegetables (salted and seasoned) in 2007 ranged from 4.6 g/1,000 kcal/day to 11.7 g/1,000 kcal/day [38]. Additionally, about 10 g/day of salted vegetable intake was estimated in China in 2002 [39]. The particularly high Korean PAFs may therefore be partly explained by the frequent consumption of kimchi, a major contributor to dietary salt and not widely eaten in Western populations.

Several studies have examined the burden of cancers attributed to low intake of vegetables and fruits. Our 2020 estimates of 1.92% for incidence and 2.34% for deaths were slightly higher than estimates from Japan (1.3% for incidence and 1.4% for deaths) [12], France (1.9% for incidence) [8], and the United States (1.3% for incidence) [6]. However, our results were lower than estimates from China (15.0% for incidence and 16.6% for deaths) [13] and the Eastern Mediterranean region (10.9% for male and 6.2% for female for incidence) [35]. The relatively low intake levels of non-starchy vegetables and fruits in our population (340.5 g/day), compared with the optimal intake range recommended by the GBD 2017 study (490-730 g/day), likely contribute to the importance of this factor in Korea’s cancer burden. Korea’s PAFs were similar to those of other high-income countries such as Japan, France, and the United States, and lower than in regions where fruit and vegetable intake remains limited. These differences may reflect variations in income levels, climate, and agricultural systems that influence food availability.

The estimated burden of cancers attributable to low dietary fiber intake (1.90% for incidence and 1.04% for deaths) fell within the range reported by other countries: 0.9% in the United States [40], 0.7% in Canada (Alberta) [41], 0.8% in Brazil [11], 3.3% in the United Kingdom [7], 1.4% in France [8], 3.3% in Germany [9], and 2.3% in Australia for incident cases [42], as well as 1.7% in Australia for deaths [10]. Our estimates may be partly explained by the relatively high prevalence (42.4%) of dietary fiber intake below the optimal range of 19-28 g/day recommended by the GBD 2017 study.

In our study, the PAFs for red meat and processed meat were 0.10% and 0.02% for incidence, and 0.49% and 0.02% for deaths, respectively. These estimates were generally lower than those from other countries, where PAFs ranged from 0.4% to 0.6% for red meat and 1.0% to 2.1% for processed meat in the United States [6], France [8], and Germany [9]. The PAFs for combined red and processed meat intake were reported as 2.7% in the United Kingdom [37], and 2.3% in Australia [43]. The lower PAFs in our study reflect the comparatively lower intakes of these meats: 61.6 g/day of red meat and 4.2 g/day of processed meat in Korea (2005), compared with 100.5 g/day and 43.5 g/day in the United States (2011-2014) [6], 68.6 g/day for red meat and 36.5 g/day for processed meat in France (in 2006) [8], 36.9 g/day for red meat and 45.7 g/day for processed meat in Germany (in 2008-2011) [9], 121 g/day (male) and 64 g/day (female) for red and processed meat in Australia (in 1995) [43], and 118 g/day (male) and 69 g/day (female) for red and processed meat in the United Kingdom (in 2000-2001) [37]. For cancers attributable to high intake of salted fish, the PAF was very small (0.01%), reflecting the low intake level of salted fish (average 3.2 g/day in 2005). Given the limited number of studies evaluating cancer burden from salted fish consumption, further research is needed.

The PAF for cancer in 2020 highlighted substantial contributions from high salted vegetable intake and low non-starchy vegetable and fruit intake. In Korea, site-specific cancer trends differ, with stomach cancer showing a steady decline, whereas colorectal and breast cancers have continued to rise [44]. These trends provide important context for interpreting the projected PAFs for dietary factors. Projections to 2030 indicated a decrease in the PAF for high salted vegetable intake, while only minimal change was expected for the PAF associated with low intake of non-starchy vegetables and fruits. Individual food items, such as kimchi and other fermented vegetables, warrant further investigation to clarify their potential role in cancer risk, given their complex balance of possible beneficial and adverse effects. Continued efforts to reduce salted vegetable intake and to increase vegetable consumption therefore remain essential for cancer prevention. Moreover, although high intakes of processed meat contributed relatively little to the cancer PAF in Korea in 2015, projections suggested a steady increase through 2030. This emphasizes the need for ongoing surveillance of processed meat intake as part of broader cancer prevention strategies.

As strengths of our study, we used nationally representative data to estimate the prevalence and average intake levels of dietary factors. We also derived pooled RRs by incorporating additional findings from available Korean cohort studies. In addition, we applied a widely used Monte Carlo simulation approach to account for statistical uncertainty. However, several limitations should be noted.

First, dietary intake based on a single 24-hour recall may not adequately represent usual intake or long-term dietary patterns and may introduce non-differential misclassification. Second, several dietary factors—such as dairy products, calcium supplements, and coffee—were not included, potentially leading to an underestimation of the diet-related cancer burden. Third, the limited number of Korean cohort studies constrains the robustness of pooled RRs and the generalizability of the findings to the entire Korean population. Fourth, our PAF estimates assumed independent effects of dietary factors, which may underestimate potential combined or synergistic effects. Fifth, excluding studies with high heterogeneity may reduce bias but may further limit generalizability. Sixth, applying a uniform 15-year latency period across all cancers may not capture site-specific latency differences. Seventh, PAF estimates may vary by age, as both dietary exposures and cancer risks differ across age groups. Finally, projections beyond 2020 relied on extrapolating historical trends and therefore may not reflect unexpected changes in screening behaviors, treatment practices, or dietary patterns.

In conclusion, an estimated 6.08% of cancer incidence and 5.70% of cancer deaths in Korea in 2020 were attributable to dietary factors, with high salted vegetable intake and low non-starchy vegetable and fruit intake as major contributors. Projections for 2030 suggest a decline in the PAF associated with high salted vegetable intake and little change in the PAF related to low non-starchy vegetable and fruit intake, though these estimates may be conservative because they rely on single-day dietary assessments. These findings underscore the importance of continued efforts to reduce salted vegetable intake, increase vegetable consumption, and monitor processed meat intake to strengthen cancer prevention. They also suggest that enhancing nutrition education, refining dietary guidelines, and exploring supportive policy measures could be valuable directions for future public health efforts.

## Figures and Tables

**Figure f4-epih-47-e2025065:**
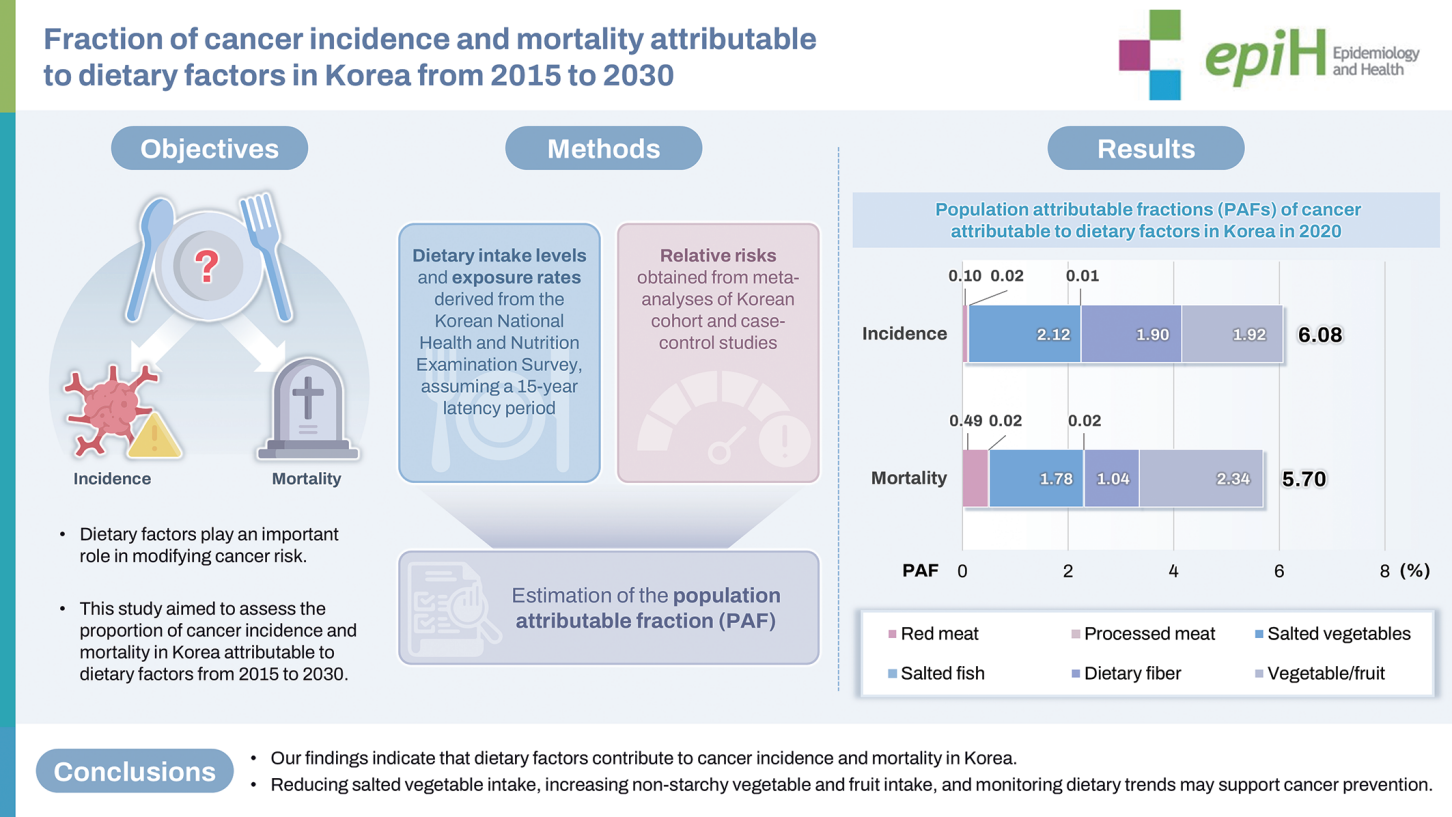


**Table 1.** PAFs^1^ of cancer attributable to dietary factors in Korea from 2015 to 2030

Dietary factors	2015		2020		2025		2030	
	PAF	AC	PAF	AC	PAF	AC	PAF	AC
Incidence								
Risk-increasing factors								
Red meat	0.08	167	0.10	240	0.10	306	0.10	345
Processed meat	0.01	12	0.02	46	0.03	96	0.08	299
Salted vegetables	3.00	6,469	2.12	5,231	1.59	4,770	1.17	4,187
Salted fish	0.02	50	0.01	36	0.01	33	0.01	29
All the above factors	3.11	6,698	2.25	5,554	1.73	5,206	1.36	4,860
Risk-decreasing factors								
Dietary fiber	1.67	3,591	1.90	4,685	1.54	4,636	1.61	5,766
Vegetable/fruit^2^	2.22	4,781	1.92	4,734	1.95	5,851	1.92	6,871
All the above factors	3.88	8,372	3.82	9,420	3.49	10,487	3.52	12,636
All dietary factors	6.99	15,070	6.08	14,973	5.23	15,693	4.88	17,496
Death								
Risk-increasing factors								
Red meat	0.45	347	0.49	404	0.71	626	0.70	656
Processed meat	0.01	5	0.02	18	0.05	44	0.15	137
Salted vegetables	2.44	1,866	1.78	1,461	1.29	1,147	0.92	865
Salted fish	0.02	19	0.02	14	0.01	11	0.01	8
All the above factors	2.92	2,237	2.31	1,897	2.06	1,829	1.78	1,666
Risk-decreasing factors								
Dietary fiber	1.05	806	1.04	856	1.16	1,025	1.29	1,209
Vegetable/fruit^2^	2.70	2,070	2.34	1,922	2.70	2,396	2.84	2,659
All the above factors	3.75	2,877	3.39	2,778	3.86	3,421	4.13	3,868
All dietary factors	6.67	5,114	5.70	4,675	5.92	5,250	5.91	5,533

PAF, population-attributable fraction; AC, attributable case.

1 The PAF for each year was estimated using the number of cancer cases in the population for the year, along with consistent relative risks and a 15-year latency period, and the prevalence of dietary factors in 2000, 2005, 2010, and 2015, respectively.

2 Vegetable/fruit means non-starchy vegetable and fruit.

**Table 2.** PAFs^1^ of diet-related cancer attributable to all dietary factors in Korea from 2015 to 2030

Variables	2015		2020		2025		2030	
	PAF	AC	PAF	AC	PAF	AC	PAF	AC
Incidence								
Nasopharynx	8.17	35	6.97	29	7.54	32	7.53	32
Stomach	27.58	8,105	24.61	6,562	22.84	6,354	20.66	5,853
Colorectal	18.75	5,084	18.38	6,491	19.34	6,802	20.03	8,686
Aerodigestive^2^	5.87	1,846	5.12	1,892	5.74	2,504	5.81	2,925
All cancers	6.99	15,070	6.08	14,973	5.23	15,693	4.88	17,496
Death								
Nasopharynx	8.89	15	7.43	12	7.98	16	7.59	18
Stomach	27.32	2,329	24.27	1,822	22.49	1,519	20.31	1,201
Colorectal	18.58	1,542	18.57	1,647	20.78	2,206	21.28	2,622
Aerodigestive^2^	6.02	1,228	5.46	1,194	6.24	1,510	6.46	1,690
All cancers	6.67	5,114	5.70	4,675	5.92	5,250	5.91	5,533

PAF, population-attributable fraction; AC, attributable case.

1 The PAF for each year was estimated using the number of cancer cases in the population for the year, along with consistent relative risks and a 15-year latency period, and the prevalence of dietary factors in 2000, 2005, 2010, and 2015, respectively.

2 Aerodigestive and some other cancers (including lung cancers) without nasopharyngeal, colorectal, and stomach cancers [C00-C10, C12-C15, C30-34].

**Table 3.** PAFs of cancer attributable to dietary factors in Korea in 2020 based on Korean, Asian, and global RR

Dietary factors	All		Male		Female	
	PAF	AC	PAF	AC	PAF	AC
Incidence						
Observed number of cancer cases	246,436		129,839		116,597	
Korean RR						
Risk-increasing factors	2.25	5,554	3.13	4,066	1.28	1,488
Risk-decreasing factors	3.82	9,420	5.30	6,883	2.18	2,537
All dietary factors	6.08	14,973	8.43	10,949	3.45	4,024
Asian RR						
Risk-increasing factors	2.34	5,773	3.19	4,143	1.40	1,629
Risk-decreasing factors	2.64	6,512	2.65	3,442	2.63	3,070
All dietary factors	4.99	12,285	5.84	7,568	4.03	4,699
Global RR						
Risk-increasing factors	1.64	4,045	2.06	2,673	1.18	1,373
Risk-decreasing factors	2.12	5,218	3.04	3,945	1.09	1,273
All dietary factors	3.76	9,263	5.10	6,617	2.27	2,646
Deaths						
Observed number of cancer deaths	82,036		50,705		31,331	
Korean RR						
Risk-increasing factors	2.31	1,897	2.76	1,400	1.59	497
Risk-decreasing factors	3.39	2,778	5.17	2,623	0.49	155
All dietary factors	5.70	4,675	7.93	4,023	2.08	652
Asian RR						
Risk-increasing factors	2.30	1,891	2.76	1,400	1.57	491
Risk-decreasing factors	1.91	1,563	2.41	1,223	1.09	340
All dietary factors	4.21	3,454	5.17	2,623	2.65	832
Global RR						
Risk-increasing factors	1.67	1,369	2.04	1,034	1.07	336
Risk-decreasing factors	3.42	2,803	5.02	2,544	0.82	258
All dietary factors	5.09	4,172	7.06	3,578	1.90	594

PAF, population-attributable fraction; AC, attributable case; RR, relative risk.

## Supplementary materials

Supplementary Material 1. Cancers caused by dietary factors

Supplementary Material 2. Age-standardized prevalence rates of dietary factors from 2000 to 2030

Supplementary Material 3. Prevalence rates of dietary factors in Korea

Supplementary Material 4. Relative risks used for the estimation of population attributable fractions

Supplementary Material 5. Population Attributable fraction (PAF) of cancer attributable to dietary factors by sex from 2015 to 2030

Supplementary Material 6. Population Attributable fraction (PAF) of diet-related cancer attributable to all dietary factors by sex from 2015 to 2030

Supplementary Material 7. Population attributable fraction (PAF) of cancer attributable to dietary factors in 2020 by dietary intake years 2000-2015

Supplementary Material 8. Cohort and case-control studies included in the meta-analysis for dietary factors and specific cancer risk
