# Supplementary material for: Fraction of cancer incidence and mortality attributable to dietary factors in Korea from 2015 to 2030
Source: Epidemiol Health. 2025 Dec 8;47:e2025065. doi: 10.4178/epih.e2025065 (PMC12884019; doi:10.4178/epih.e2025065)
Supplement: Supplementary Material 1. — Cancers caused by dietary factors [file epih-47-e2025065-Supplementary-1.docx]

Supplementary Material 1. Cancers caused by dietary factors

| **Dietary factors** | **Cancers** | **Selection criteria** |
| --- | --- | --- |
| ***Risk-increasing dietary factors*** |  |  |
| Red meat (High intake) | Colorectal (C18-C20) | WCRF CUP: Probable |
| Processed meat (High intake) | Colorectal (C18-C20) | WCRF CUP: Convincing  IARC Group 1: Carcinogenic to humans |
| Salted vegetables (High intake) | Stomach (C16) | WCRF CUP: Probable |
| Salted fish (High intake) | Nasopharyngeal (C11)  Stomach (C16) | WCRF CUP: Probable  IARC Group 1: Carcinogenic to humans |
| ***Risk-decreasing dietary factors*** |  |  |
| Dietary fiber (Low intake) | Colorectal (C18-C20) | WCRF CUP: Probable |
| Non-starch vegetable and fruit (Low intake) | Aerodigestive and some other (aggregated)^1^  (C00-C16, C18-C20, C30-34) | WCRF CUP: Probable |

WCRF, world cancer research fund; CUP, the continuous update project; IARC, international agency for research on cancer

^1^Some other cancer included colorectal, stomach, and lung cancer
