# Supplementary material for: Fraction of cancer incidence and mortality attributable to dietary factors in Korea from 2015 to 2030
Source: Epidemiol Health. 2025 Dec 8;47:e2025065. doi: 10.4178/epih.e2025065 (PMC12884019; doi:10.4178/epih.e2025065)
Supplement: Supplementary Material 2. — Age-standardized prevalence rates of dietary factors from 2000 to 2030 [file epih-47-e2025065-Supplementary-2.docx]

**Red meat prevalence (%) Processed meat prevalence (%)**

**Dietary fiber prevalence (%) Salted vegetables prevalence (%)**

**Salted fish prevalence (%) Non-starch vegetable and fruit prevalence (%)**

**Supplementary Material 2. Age-standardized prevalence rates of dietary factors from 2000 to 2030.**

Blue, for male; Orange, for female; Green, for total population. Source: Korea National Health and Nutritional Examination Survey 2001, 2005, and 2007-2018
