# Supplementary material for: Fraction of cancer incidence and mortality attributable to dietary factors in Korea from 2015 to 2030
Source: Epidemiol Health. 2025 Dec 8;47:e2025065. doi: 10.4178/epih.e2025065 (PMC12884019; doi:10.4178/epih.e2025065)
Supplement: Supplementary Material 3. — Prevalence rates of dietary factors in Korea [file epih-47-e2025065-Supplementary-3.docx]

**Supplementary Material 3. Prevalence rates and mean intake levels of dietary factors in Korea**

| **Year** |  | **Sex** | **Red meat^1^** | **Processed meat^1^** | **Salted vegetables^1^** | **Salted fish^1^** | **Dietary fiber^2,3^** | **Non-starch vegetable and fruit^2^** |
| --- | --- | --- | --- | --- | --- | --- | --- | --- |
| 2000^4^ | Prevalence  Rates (%) | Male | 52.6 | 4.4 | 99.0 | 9.7 | 37.6 | 79.2 |
|  |  | Female | 36.7 | 4.7 | 95.8 | 8.6 | 48.3 | 76.7 |
|  | Mean intake level (g/d) | Male | 73.9 | 3.5 | 186.8 | 4.7 | 24.5 | 333.9 |
|  |  | Female | 41.7 | 2.3 | 146.3 | 3.6 | 21.2 | 336.0 |
| 2005^5^ | Prevalence  Rates (%) | Male | 54.4 | 9.7 | 96.4 | 7.0 | 36.4 | 85.2 |
|  |  | Female | 40.6 | 9.0 | 93.0 | 7.9 | 48.3 | 86.6 |
|  | Mean intake level (g/d) | Male | 76.3 | 5.2 | 170.9 | 4.7 | 24.9 | 278.7 |
|  |  | Female | 47.6 | 3.2 | 126.2 | 4.0 | 21.4 | 263.2 |
| 2010^5^ | Prevalence  Rates (%) | Male | 58.3 | 14.1 | 93.7 | 10.2 | 35.2 | 75.4 |
|  |  | Female | 42.7 | 13.1 | 89.0 | 9.7 | 48.2 | 73.2 |
|  | Mean intake level (g/d) | Male | 94.5 | 6.9 | 153.5 | 3.0 | 25.4 | 359.7 |
|  |  | Female | 49.0 | 5.0 | 108.0 | 2.5 | 21.6 | 365.0 |
| 2015^5^ | Prevalence  Rates (%) | Male | 57.5 | 21.0 | 91.3 | 6.6 | 34.5 | 73.5 |
|  |  | Female | 44.4 | 19.8 | 84.6 | 6.7 | 49.3 | 73.4 |
|  | Mean intake level (g/d) | Male | 83.1 | 12.5 | 136.6 | 4.7 | 25.9 | 381.8 |
|  |  | Female | 50.5 | 7.8 | 90.4 | 1.3 | 21.7 | 379.4 |
| 2020^4^ | Prevalence  Rates (%) | Male | 62.5 | 26.0 | 88.4 | 7.9 | 32.7 | 75.4 |
|  |  | Female | 46.7 | 23.8 | 81.6 | 7.3 | 48.2 | 75.0 |
|  | Mean intake level (g/d) | Male | 100.0 | 15.1 | 121.5 | 1.8 | 26.3 | 358.6 |
|  |  | Female | 52.4 | 10.4 | 77.2 | 1.1 | 22.1 | 352.8 |
| 2025^4^ | Prevalence  Rates (%) | Male | 64.9 | 31.4 | 85.8 | 7.4 | 31.5 | 74.4 |
|  |  | Female | 49.1 | 28.6 | 78.1 | 7.0 | 48.1 | 74.5 |
|  | Mean intake level (g/d) | Male | 107.8 | 21.8 | 109.2 | 1.4 | 26.8 | 365.1 |
|  |  | Female | 55.4 | 15.0 | 65.8 | 0.8 | 22.3 | 357.2 |
| 2030^4^ | Prevalence  Rates (%) | Male | 67.4 | 36.8 | 83.2 | 7.0 | 30.2 | 73.5 |
|  |  | Female | 51.6 | 33.3 | 74.5 | 6.7 | 48.1 | 74.1 |
|  | Mean intake level (g/d) | Male | 116.3 | 31.4 | 98.0 | 1.1 | 27.3 | 371.7 |
|  |  | Female | 58.7 | 21.8 | 56.1 | 0.6 | 22.5 | 361.6 |

The prevalence rates and mean intake levels were age-standardized using the census 2000

^1^Cut-off was used by average intake values of reference groups among published global studies (salted vegetables: ≥9 g/day, salted fish: ≥3 g/day).

^2^Cut-off was used by optimal intake suggested form the Global Burden of Disease study 2017 (red meat: >27 g/day, processed meat: >4 g/day, dietary fiber: <19 g/day, non-starch vegetables and fruits: <490 g/day).

^3^We estimated the prevalence rates and mean intake levels in 2000, 2020, 2025, and 2030 using the KNHANES (Korea National Health and Nutrition Examination Survey) data from 2013-2018

^4^We estimated the prevalence rates and mean intake levels in 2000, 2020, 2025, and 2030 using a linear regression model with the KNHANES data from 2001, 2005, and 2007-2018.

^5^We estimated the prevalence rates and mean intake levels in 2005, 2010, and 2015 using the KNHANES data from each respective year.
