# Supplementary material for: Fraction of cancer incidence and mortality attributable to dietary factors in Korea from 2015 to 2030
Source: Epidemiol Health. 2025 Dec 8;47:e2025065. doi: 10.4178/epih.e2025065 (PMC12884019; doi:10.4178/epih.e2025065)
Supplement: Supplementary Material 4. — Relative risks used for the estimation of population attributable fractions [file epih-47-e2025065-Supplementary-4.docx]

Supplementary Material 4. Relative risks used for the estimation of population attributable fractions

|  |  | **Incidence^1^** | | | | **Death^2^** | | | |
| --- | --- | --- | --- | --- | --- | --- | --- | --- | --- |
|  |  | **Male** | | **Female** | | **Male** | | **Female** | |
| ***Korean Cohort and Case-control Studies^3^*** |  |  |  |  |  |  |  |  |  |
| ***Risk-increasing dietary factors*** |  | **Study** | **RR (95% CI)** | **Study** | **RR (95% CI)** | **Study** | **RR (95% CI)** | **Study** | **RR (95% CI)** |
| Red meat (per 120 g/day) | Colorectal (C18-C20) | 3 | 1.03 (0.71-1.48)^CK^ | 3 | 1.03 (0.71-1.48)^CK^ | 2 | 1.22 (0.91-1.64)^EK^ | 2 | 1.10 (0.63-1.91)^EK^ |
| Processed meat (per 50 g/day) | Colorectal (C18-C20) | 2 | 1.12 (0.70-1.77)^EA^ | 2 | 1.31 (1.04-1.65)^EA^ | 2 | 1.35 (0.81-2.24)^EA^ | 2 | 1.15 (0.60-2.20)^EA^ |
| Salted vegetables (per 40 g/day) | Stomach (C16) | 10 | 1.06 (0.95-1.18)^CA^ | 10 | 1.06 (0.95-1.18)^CA^ | 10 | 1.06 (0.95-1.18)^CA,6^ | 10 | 1.06 (0.95-1.18)^CA,6^ |
| Salted fish (per 20 g/day) | Nasopharyngeal (C11) | 2 | 2.18 (1.50-3.16)^EA^ | 1 | 2.82 (0.71-11.26)^EA^ | 2 | 2.18 (1.50-3.16)^EA,6^ | 1 | 2.82 (0.71-11.26)^EA,6^ |
|  | Stomach (C16) | 5 | 1.07 (0.90-1.27)^CA^ | 5 | 1.07 (0.90-1.27)^CA^ | 3 | 1.07 (0.93-1.23)^EK^ | 2 | 1.15 (0.75-1.78)^EK^ |
| ***Risk-decreasing dietary factors*** |  | **Study** | **RR (95% CI)** | **Study** | **RR (95% CI)** | **Study** | **RR (95% CI)** | **Study** | **RR (95% CI)** |
| Dietary fiber (per 10 g/day) | Colorectal (C18-C20) | 3 | 0.89 (0.66-1.20)^EA^ | 5 | 0.85 (0.71-1.02)^EA^ | 2 | 0.86 (0.56-1.32)^EK^ | 2 | 0.97 (0.58-1.62)^EK^ |
| Non-starch vegetable and fruit (per 80 g/day) | Aerodigestive | 2 | 0.98 (0.91-1.05)^EK^ | 2 | 0.999 (0.89-1.13)^EK^ | 2 | 0.98 (0.91-1.05)^EK,6^ | 2 | 0.999 (0.89-1.13)^EK,6^ |
|  | (C00-C16, C18-C20, C30-34) |  |  |  |  |  |  |  |  |
| ***Asian Cohort and Case-control Studies^4^*** |  |  |  |  |  |  |  |  |  |
| ***Risk-increasing dietary factors*** |  | **Study** | **RR (95% CI)** | **Study** | **RR (95% CI)** | **Study** | **RR (95% CI)** | **Study** | **RR (95% CI)** |
| Red meat (per 120 g/day) | Colorectal (C18-C20) | 4 | 1.04 (0.72-1.49)^EA^ | 4 | 1.09 (0.87-1.36)^EA^ | 2 | 1.22 (0.91-1.64)^EA^ | 2 | 1.10 (0.63-1.91)^EA^ |
| Processed meat (per 50 g/day) | Colorectal (C18-C20) | 2 | 1.12 (0.70-1.77)^EA^ | 2 | 1.31 (1.04-1.65)^EA^ | 2 | 1.35 (0.81-2.24)^EA^ | 2 | 1.15 (0.60-2.20)^EA^ |
| Salted vegetables (per 40 g/day) | Stomach (C16) | 10 | 1.06 (0.95-1.18)^CA^ | 10 | 1.06 (0.95-1.18)^CA^ | 10 | 1.06 (0.95-1.18)^CA,6^ | 10 | 1.06 (0.95-1.18)^CA,6^ |
| Salted fish (per 20 g/day) | Nasopharyngeal (C11) | 2 | 2.18 (1.50-3.16)^EA^ | 1 | 2.82 (0.71-11.26)^EA^ | 2 | 2.18 (1.50-3.16)^EA,6^ | 1 | 2.82 (0.71-11.26)^EA,6^ |
|  | Stomach (C16) | 5 | 1.07 (0.90-1.27)^CA^ | 5 | 1.07 (0.90-1.27)^CA^ | 5 | 1.07 (1.004-1.13)^EA^ | 4 | 1.002 (0.90-1.11)^EA^ |
| ***Risk-decreasing dietary factors*** |  | **Study** | **RR (95% CI)** | **Study** | **RR (95% CI)** | **Study** | **RR (95% CI)** | **Study** | **RR (95% CI)** |
| Dietary fiber (per 10 g/day) | Colorectal (C18-C20) | 3 | 0.89 (0.66-1.20)^EA^ | 5 | 0.85 (0.71-1.02)^EA^ | 2 | 0.86 (0.56-1.32)^EA^ | 2 | 0.97 (0.58-1.62)^EA^ |
| Non-starch vegetable and fruit (per 80 g/day) | Aerodigestive | 7 | 0.99 (0.97-1.02)^CA^ | 7 | 0.99 (0.97-1.02)^CA^ | 7 | 0.99 (0.97-1.02)^CA,6^ | 7 | 0.99 (0.97-1.02)^CA,6^ |
|  | (C00-C16, C18-C20, C30-34) |  |  |  |  |  |  |  |  |
| ***Global Cohort Studies^5^*** |  |  |  |  |  |  |  |  |  |
| ***Risk-increasing dietary factors*** |  | **Study** | **RR (95% CI)** | **Study** | **RR (95% CI)** | **Study** | **RR (95% CI)** | **Study** | **RR (95% CI)** |
| Red meat (per 120 g/day) | Colorectal (C18-C20) | 7 | 1.02 (0.82-1.27)^EG^ | 10 | 1.21 (1.07-1.38)^EG^ | 2 | 1.22 (0.91-1.64)^EG^ | 2 | 1.10 (0.63-1.91)^EG^ |
| Processed meat (per 50 g/day) | Colorectal (C18-C20) | 8 | 1.07 (0.96-1.19)^EG^ | 13 | 1.27 (1.15-1.39)^EG^ | 2 | 1.35 (0.81-2.24)^EG^ | 2 | 1.15 (0.60-2.20)^EG^ |
| Salted vegetables (per 40 g/day) | Stomach (C16) | 6 | 1.04 (0.96-1.12)^CG^ | 6 | 1.04 (0.96-1.12)^CG^ | 6 | 1.04 (0.96-1.12)^CG,6^ | 6 | 1.04 (0.96-1.12)^CG,6^ |
| Salted fish (per 20 g/day) | Nasopharyngeal (C11) | 2 | 2.18 (1.50-3.16)^EA^ | 1 | 2.82 (0.71-11.26)^EA^ | 2 | 2.18 (1.50-3.16)^EA,6^ | 1 | 2.82 (0.71-11.26)^EA,6^ |
|  | Stomach (C16) | 3 | 1.03 (0.92-1.15)^CG^ | 3 | 1.03 (0.92-1.15)^CG^ | 5 | 1.07 (1.004-1.13)^EG^ | 4 | 1.002 (0.90-1.11)^EG^ |
| ***Risk-decreasing dietary factors*** |  | **Study** | **RR (95% CI)** | **Study** | **RR (95% CI)** | **Study** | **RR (95% CI)** | **Study** | **RR (95% CI)** |
| Dietary fiber (per 10 g/day) | Colorectal (C18-C20) | 10 | 0.94 (0.92-0.97)^EG^ | 15 | 0.94 (0.90-0.98)^EG^ | 3 | 0.75 (0.59-0.95)^EG^ | 2 | 0.97 (0.58-1.62)^EG^ |
| Non-starch vegetable and fruit (per 80 g/day) | Aerodigestive | 14 | 0.99 (0.97-0.997)^EG^ | 15 | 0.996 (0.99-1.01)^EG^ | 14 | 0.99 (0.97-0.997)^EG,6^ | 15 | 0.996 (0.99-1.01)^EG,6^ |
|  | (C00-C16, C18-C20, C30-34) |  |  |  |  |  |  |  |  |

RR, relative risk; CI, confidence interval
Cohort and case-control studies included in the meta-analysis of dietary factors and specific cancer risk are listed in Supplementary Material 8.
^1^Meta-analyzed after calculating RR on cancer incidence from raw data of 2 Korean cohort studies (KMCC; NWS/DGS)

^2^Meta-analyzed after calculating RR on cancer death from raw data of 4 Korean cohort studies (KMCC; NWS/DGS; KoGES; KNHANES)

^3^The prioritization of RRs selected for estimating PAFs was structured in the following order: 1) RRs pooled from each sex in Korean cohort and case-control studies (EK); 2) RRs pooled from the combined sexes in Korean cohort and case-control studies (CK); 3) RRs pooled from each sex in Asian cohort and case-control studies (EA); 4) RRs pooled from the combined sexes in Asian cohort and case-control studies (CA); 5) RRs pooled from each sex in global cohort studies (EG); 6) RRs pooled from the combined sexes in global cohort studies (CG).

^4^The prioritization of RRs selected for estimating PAFs was structured in the following order: 1) RRs pooled from each sex in Asian cohort and case-control studies (EA); 2) RRs pooled from the combined sexes in Asian cohort and case-control studies (CA); 3) RRs pooled from each sex in global cohort studies (EG); 4) RRs pooled from the combined sexes in global cohort studies (CG).

^5^The prioritization of RRs selected for estimating PAFs was structured in the following order: 1) RRs pooled from each sex in global cohort studies (EG); 2) RRs pooled from the combined sexes in global cohort studies (CG); 3) RRs pooled from each sex in Asian cohort and case-control studies (EA); 4) RRs pooled from the combined sexes in Asian cohort and case-control studies (CA).

^6^RRs for incidence were used instead of RRs for mortality due to their unavailability.
