# Supplementary material for: Fraction of cancer incidence and mortality attributable to dietary factors in Korea from 2015 to 2030
Source: Epidemiol Health. 2025 Dec 8;47:e2025065. doi: 10.4178/epih.e2025065 (PMC12884019; doi:10.4178/epih.e2025065)
Supplement: Supplementary Material 5. — Population Attributable fraction (PAF) of cancer attributable to dietary factors by sex from 2015 to 2030 [file epih-47-e2025065-Supplementary-5.docx]

Supplementary Material 5. Population Attributable fraction (PAF) of cancer attributable to dietary factors by sex from 2015 to 2030

|  | **Male** | | | | | | | | **Female** | | | | | | | |
| --- | --- | --- | --- | --- | --- | --- | --- | --- | --- | --- | --- | --- | --- | --- | --- | --- |
|  | **2015** | | **2020** | | **2025** | | **2030** | | **2015** | | **2020** | | **2025** | | **2030** | |
|  | PAF | AC | PAF | AC | PAF | AC | PAF | AC | PAF | AC | PAF | AC | PAF | AC | PAF | AC |
| **Incidence** |  |  |  |  |  |  |  |  |  |  |  |  |  |  |  |  |
| ***Risk-increasing factors*** |  |  |  |  |  |  |  |  |  |  |  |  |  |  |  |  |
| Red meat | 0.12 | 132 | 0.14 | 181 | 0.16 | 244 | 0.15 | 264 | 0.03 | 36 | 0.05 | 59 | 0.04 | 62 | 0.05 | 81 |
| Processed meat | 0.005 | 5 | 0.02 | 23 | 0.03 | 45 | 0.08 | 153 | 0.01 | 7 | 0.02 | 23 | 0.03 | 51 | 0.08 | 146 |
| Salted vegetables | 4.10 | 4,665 | 2.96 | 3,837 | 2.30 | 3,566 | 1.77 | 3, 199 | 1.77 | 1,803 | 1.20 | 1,394 | 0.83 | 1,204 | 0.55 | 988 |
| Salted fish | 0.03 | 38 | 0.02 | 25 | 0.02 | 24 | 0.01 | 25 | 0.01 | 12 | 0.01 | 11 | 0.01 | 9 | 0.002 | 3 |
| **All factors above** | **4.26** | **4,841** | **3.13** | **4,066** | **2.51** | **3,879** | **2.02** | **3,642** | **1.82** | **4**,**053** | **1.28** | **1**,**488** | **0.91** | **1**,**326** | **0.68** | **1**,**218** |
|  |  |  |  |  |  |  |  |  |  |  |  |  |  |  |  |  |
| ***Risk-decreasing factors*** |  |  |  |  |  |  |  |  |  |  |  |  |  |  |  |  |
| Dietary fiber | 1.54 | 1,748 | 1.72 | 2,229 | 1.45 | 2,236 | 1.55 | 2,798 | 1.81 | 1,843 | 2.11 | 2,456 | 1.65 | 2,400 | 1.66 | 2,968 |
| Vegetable/fruit^1^ | 4.13 | 4,703 | 3.58 | 4,654 | 3.72 | 5,750 | 3.74 | 6,748 | 0.08 | 78 | 0.07 | 80 | 0.07 | 101 | 0.07 | 123 |
| **All factors above** | **5.67** | **6,451** | **5.30** | **6,883** | **5.16** | **7,986** | **5.30** | **9,545** | **1.82** | **1**,**857** | **2.18** | **2**,**537** | **1.72** | **2**,**501** | **1.73** | **3**,**091** |
|  |  |  |  |  |  |  |  |  |  |  |  |  |  |  |  |  |
| ***All dietary factors*** | **9.93** | **11,291** | **8.43** | **10,949** | **7.67** | **11,865** | **7.32** | **13,187** | **3.71** | **3,778** | **3.45** | **4,024** | **2.63** | **3,828** | **2.42** | **4,309** |
| **Death** |  |  |  |  |  |  |  |  |  |  |  |  |  |  |  |  |
| ***Risk-increasing factors*** |  |  |  |  |  |  |  |  |  |  |  |  |  |  |  |  |
| Red meat | 0.64 | 303 | 0.68 | 345 | 1.01 | 551 | 0.99 | 564 | 0.15 | 44 | 0.19 | 59 | 0.22 | 75 | 0.25 | 92 |
| Processed meat | 0.01 | 4 | 0.03 | 15 | 0.07 | 36 | 0.20 | 114 | 0.004 | 1 | 0.01 | 3 | 0.02 | 8 | 0.06 | 22 |
| Salted vegetables | 2.74 | 1305 | 2.04 | 1,032 | 1.52 | 830 | 1.13 | 643 | 1.93 | 561 | 1.37 | 428 | 0.93 | 317 | 0.61 | 222 |
| Salted fish | 0.02 | 12 | 0.01 | 7 | 0.01 | 7 | 0.01 | 7 | 0.02 | 7 | 0.02 | 7 | 0.01 | 5 | 0.004 | 2 |
| **All factors above** | **3.41** | **1,624** | **2.76** | **1,400** | **2.61** | **1,423** | **2.32** | **1,328** | **2.11** | **613** | **1.59** | **497** | **1.18** | **405** | **0.92** | **338** |
|  |  |  |  |  |  |  |  |  |  |  |  |  |  |  |  |  |
| ***Risk-decreasing factors*** |  |  |  |  |  |  |  |  |  |  |  |  |  |  |  |  |
| Dietary fiber | 1.45 | 688 | 1.44 | 729 | 1.60 | 874 | 1.81 | 1.032 | 0.41 | 118 | 0.41 | 127 | 0.44 | 151 | 0.48 | 176 |
| Vegetable/fruit^1^ | 4.29 | 2039 | 3.74 | 1894 | 4.33 | 2,360 | 4.58 | 2,620 | 0.11 | 31 | 0.09 | 28 | 0.10 | 36 | 0.11 | 39 |
| **All factors above** | **5.74** | **2,727** | **5.17** | **2,623** | **5.94** | **3,235** | **6.39** | **3,652** | **0.51** | **149** | **0.49** | **155** | **0.55** | **187** | **0.59** | **216** |
|  |  |  |  |  |  |  |  |  |  |  |  |  |  |  |  |  |
| ***All dietary factors*** | **9.15** | **4,351** | **7.93** | **4023** | **8.55** | **4,658** | **8.72** | **4,979** | **2.62** | **763** | **2.08** | **652** | **1.73** | **592** | **1.51** | **552** |

PAF, population attributable fraction; AC, attributable case

The PAF for each year was estimated using the number of cancer cases in the population for the year, along with consistent RRs and a 15-year latency period, and the prevalence of dietary factors in 2000, 2005, 2010, and 2015, respectively.
^1^Vegetable/fruit means non-starchy vegetable and fruit.
