# Supplementary material for: Fraction of cancer incidence and mortality attributable to dietary factors in Korea from 2015 to 2030
Source: Epidemiol Health. 2025 Dec 8;47:e2025065. doi: 10.4178/epih.e2025065 (PMC12884019; doi:10.4178/epih.e2025065)
Supplement: Supplementary Material 6. — Population Attributable fraction (PAF) of diet-related cancer attributable to all dietary factors by sex from 2015 to 2030 [file epih-47-e2025065-Supplementary-6.docx]

Supplementary Material 6. Population Attributable fraction (PAF) of diet-related cancer attributable to all dietary factors by sex from 2015 to 2030

|  | **Male** | | | | | | | | **Female** | | | | | | | |
| --- | --- | --- | --- | --- | --- | --- | --- | --- | --- | --- | --- | --- | --- | --- | --- | --- |
|  | **2015** | | **2020** | | **2025** | | **2030** | | **2015** | | **2020** | | **2025** | | **2030** | |
|  | PAF | AC | PAF | AC | PAF | AC | PAF | AC | PAF | AC | PAF | AC | PAF | AC | PAF | AC |
| **Incidence** |  |  |  |  |  |  |  |  |  |  |  |  |  |  |  |  |
| Nasopharynx | 9.78 | 32 | 8.43 | 27 | 9.41 | 31 | 9.71 | 31 | 2.42 | 2 | 2.28 | 2 | 1.30 | 1 | 0.29 | 0 |
| Stomach | 31.83 | 6,266 | 28.75 | 5,137 | 27.36 | 5,117 | 25.36 | 4,835 | 18.97 | 1,839 | 16.20 | 1,425 | 13.57 | 1,237 | 10.98 | 1,018 |
| Colorectal | 19.68 | 3,168 | 18.88 | 3,919 | 20.17 | 4,251 | 20.70 | 5,442 | 17.39 | 1,916 | 17.67 | 2,572 | 18.11 | 2,552 | 18.98 | 3,244 |
| Aerodigestive^1^ | 7.97 | 1,822 | 7.16 | 1,866 | 8.19 | 2,467 | 8.47 | 2,878 | 0.27 | 23 | 0.23 | 25 | 0.27 | 37 | 0.29 | 47 |
|  |  |  |  |  |  |  |  |  |  |  |  |  |  |  |  |  |
| ***All cancers*** | **9.93** | **11,291** | **8.43** | **10,949** | **7.67** | **11,865** | **7.32** | **13,187** | **3.71** | **3,778** | **3.45** | **4,024** | **2.63** | **3,828** | **2.42** | **4,309** |
| **Death** |  |  |  |  |  |  |  |  |  |  |  |  |  |  |  |  |
| Nasopharynx | 10.19 | 14 | 8.76 | 11 | 9.48 | 15 | 9.55 | 18 | 3.40 | 1 | 2.94 | 1 | 2.60 | 1 | 0.29 | 0 |
| Stomach | 31.83 | 1,753 | 28.75 | 1,382 | 27.38 | 1,191 | 25.36 | 972 | 19.08 | 576 | 16.29 | 440 | 13.64 | 328 | 11.01 | 229 |
| Colorectal | 29.15 | 1,369 | 28.82 | 1,449 | 32.17 | 1,960 | 32.35 | 2,317 | 4.80 | 173 | 5.16 | 198 | 5.44 | 246 | 5.91 | 305 |
| Aerodigestive^1^ | 7.97 | 1,214 | 7.16 | 1,181 | 8.19 | 1,493 | 8.47 | 1,672 | 0.27 | 14 | 0.23 | 13 | 0.27 | 17 | 0.29 | 18 |
|  |  |  |  |  |  |  |  |  |  |  |  |  |  |  |  |  |
| ***All cancers*** | **9.15** | **4,351** | **7.93** | **4023** | **8.55** | **4,658** | **8.72** | **4,979** | **2.62** | **763** | **2.08** | **652** | **1.73** | **592** | **1.51** | **552** |

PAF, population attributable fraction; AC, attributable case

The PAF for each year was estimated using the number of cancer cases in the population for the year, along with consistent RRs and a 15-year latency period, and the prevalence of dietary factors in 2000, 2005, 2010, and 2015, respectively.
^1^Aerodigestive and some other cancers (including lung cancers) without nasopharyngeal, colorectal, and stomach cancers [C00-C10, C12-C15, C30-34].
