# Supplementary material for: Fraction of cancer incidence and mortality attributable to dietary factors in Korea from 2015 to 2030
Source: Epidemiol Health. 2025 Dec 8;47:e2025065. doi: 10.4178/epih.e2025065 (PMC12884019; doi:10.4178/epih.e2025065)
Supplement: Supplementary Material 7. — Population attributable fraction (PAF) of cancer attributable to dietary factors in 2020 by dietary intake years 2000-2015 [file epih-47-e2025065-Supplementary-7.docx]

**Supplementary Material 7. Population attributable fraction (PAF) of cancer attributable to dietary factors in 2020 by dietary intake years 2000-2015**

|  | **Dietary factors** | **All** | | **Male** | | **Female** | |
| --- | --- | --- | --- | --- | --- | --- | --- |
| **Incidence** |  |  |  |  |  |  |  |
| **Observed number of**  **cancer cases** | | 246,436 | | 129,839 | | 116,597 | |
|  |  | **PAF** | **AC** | **PAF** | **AC** | **PAF** | **AC** |
| **Dietary intake in 2000** | Risk-increasing factors | 2.49 | 6,148 | 3.42 | 4,445 | 1.46 | 1,702 |
|  | Risk-decreasing factors | 4.04 | 9,962 | 5.73 | 7,437 | 2.17 | 2,525 |
|  | All dietary factors | **6.54** | **16,110** | **9.15** | **11,882** | **3.63** | **4,228** |
| **Dietary intake in 2005** | Risk-increasing factors | 2.25 | 5,554 | 3.13 | 4,066 | 1.28 | 1,488 |
|  | Risk-decreasing factors | 3.82 | 9,420 | 5.30 | 6,883 | 2.18 | 2,537 |
|  | All dietary factors | **6.08** | **14,973** | **8.43** | **10,949** | **3.45** | **4,024** |
| **Dietary intake in 2010** | Risk-increasing factors | 2.03 | 5,002 | 2.86 | 3,715 | 1.10 | 1,287 |
|  | Risk-decreasing factors | 4.10 | 10,097 | 5.80 | 7,525 | 2.21 | 2,573 |
|  | All dietary factors | **6.13** | **15,099** | **8.66** | **11,240** | **3.31** | **3,859** |
| **Dietary intake in 2015** | Risk-increasing factors | 1.82 | 4,486 | 2.58 | 3,352 | 0.97 | 1,134 |
|  | Risk-decreasing factors | 4.20 | 10,344 | 5.94 | 7,718 | 2.25 | 2,626 |
|  | All dietary factors | **6.02** | **14,830** | **8.53** | **11,070** | **3.23** | **3,760** |
| **Death** |  |  |  |  |  |  |  |
| **Observed number of**  **cancer deaths** | | 82,036 | | 50,705 | | 31,331 | |
|  |  | **PAF** | **AC** | **PAF** | **AC** | **PAF** | **AC** |
| **Dietary intake in 2000** | Risk-increasing factors | 2.48 | 2,035 | 2.92 | 1,478 | 1.78 | 557 |
|  | Risk-decreasing factors | 3.66 | 3,003 | 5.61 | 2,846 | 0.50 | 158 |
|  | All dietary factors | **6.14** | **5,038** | **8.53** | **4,324** | **2.28** | **715** |
| **Dietary intake in 2005** | Risk-increasing factors | 2.31 | 1,897 | 2.76 | 1,400 | 1.59 | 497 |
|  | Risk-decreasing factors | 3.39 | 2,778 | 5.17 | 2,623 | 0.49 | 155 |
|  | All dietary factors | **5.70** | **4,676** | **7.93** | **4,023** | **2.08** | **652** |
| **Dietary intake in 2010** | Risk-increasing factors | 2.24 | 1,841 | 2.78 | 1,408 | 1.38 | 433 |
|  | Risk-decreasing factors | 3.72 | 3,049 | 5.69 | 2,888 | 0.51 | 161 |
|  | All dietary factors | **5.96** | **4,889** | **8.47** | **4,295** | **1.90** | **594** |
| **Dietary intake in 2015** | Risk-increasing factors | 2.03 | 1,665 | 2.54 | 1,290 | 1.20 | 375 |
|  | Risk-decreasing factors | 3.82 | 3,132 | 5.85 | 2,966 | 0.53 | 165 |
|  | All dietary factors | **5.85** | **4,796** | **8.39** | **4,256** | **1.72** | **540** |

PAF, population attributable fraction; AC, attributable case; RR, relative risk
