# Supplementary material for: Fraction of cancer incidence and mortality attributable to dietary factors in Korea from 2015 to 2030
Source: Epidemiol Health. 2025 Dec 8;47:e2025065. doi: 10.4178/epih.e2025065 (PMC12884019; doi:10.4178/epih.e2025065)
Supplement: Supplementary Material 8. — Cohort and case-control studies included in the meta-analysis for dietary factors and specific cancer risk [file epih-47-e2025065-Supplementary-8.docx]

**Supplementary Material 8. Cohort and case-control studies included in the meta-analysis for dietary factors and specific cancer risk**

| **Author, year** | **Sex** | **Study design** | **Dietary assessment** | **Outcome** | **Exposure** | **Exposure level** | **Case/person-time or**  **Case/control no.**  **in each category** | **RR (95% CI)** | **Meta-analysis** |
| --- | --- | --- | --- | --- | --- | --- | --- | --- | --- |
| **Red meat and colorectal cancer** | | | | | | | | | |
| Islam et al., 2019 | M/W | Cohort (JPHC-5y, TAKAYAMA) | Country/centre specific dietary questionnaires | Colorectal (incidence) | Unprocessed  red meat | per 100 g/day | 3,550/123,635 | Men: 1.19 (0.75-1.89)  Women: 1.07 (0.88-1.31) | Combined, Men/Women |
| Lee et al., 2009 | W | Cohort (SWHS) | FFQ | Colorectal (incidence) | Red meat | Quintile 1  Quintile 2  Quintile 3  Quintile 4 Quintile 5 | 108/106,383 80/107,803 65/108,157 79/108,710 62/109,103 | 1.0 (ref) 0.9 0.7 1.0 0.8 (0.6-1.1) | Combined, Men/Women |
| Singh and Fraser, 1998 | Both | Cohort (AHS) | FFQ | Colon (incidence) | Red meat | Never >0 to <1 time/week ≥1 time/week | 42/58,438 40/42,211 45/63,209 | 1.00 (ref) 1.58 (1.01-2.45) 1.41 (0.90-2.21) | Combined |
| Wei et al., 2004 | M/W | Cohort (NHS, HPFS) | FFQ | Colon (incidence) | Beef, pork, or lamb as a main dish | 0 servings per day  <3 servings per month 1 serving per week 2 to 4 servings per week 5 or more servings per week  **WCRF per 100 g/day | Total: 43/Not reported 170/Not reported 333/Not reported 436/Not reported 155/Not reported  Men (HPFS): 30/Not reported 100/Not reported 139/Not reported 166/Not reported 32/Not reported  Women (NHS):  13/Not reported 70/Not reported 194/Not reported 270/Not reported 123/Not reported | Total: 1.00 (ref) 1.43 (1.02-2.02) 1.29 (0.92-1.79) 1.37 (0.98-1.90) 1.43 (1.00-2.05)  Men (HPFS): 1.00 (ref) 1.53 (1.01-2.33) 1.35 (0.89-2.03) 1.20 (0.96-2.18) 1.35 (0.80-2.27)  Women (NHS): 1.00 (ref) 1.22 (0.67-2.22) 1.16 (0.66-2.05) 1.20 (0.63-2.11) 1.31 (0.73-2.36)  **WCRF Men: 1.08 (0.73-1.59) Women: 1.18 (0.75-1.85) | Men/Women |
|  |  |  |  | Rectal (incidence) | Beef, pork, or lamb as a main dish | 0 servings per day  <3 servings per month 1 serving per week 2 to 4 servings per week 5 or more servings per week  **WCRF per 100 g/day | Total: 14/Not reported 51/Not reported 100/Not reported 142/Not reported 31/Not reported  Men (HPFS): 10/Not reported 27/Not reported 42/Not reported 49/Not reported 7/Not reported  Women (NHS):  4/Not reported 24/Not reported 58/Not reported 93/Not reported 24/Not reported | Total: 1.00 (ref) 1.28 (0.70-2.34) 1.13 (0.64-2.02) 1.30 (0.73-2.32) 0.90 (0.47-1.75)  Men (HPFS): 1.00 (ref) 1.27 (0.61-2.66) 1.15 (0.56-2.38) 1.27 (0.62-2.63) 0.90 (0.34-2.45)  Women (NHS): 1.00 (ref) 1.37 (0.47-3.97) 1.14 (0.41-1.37) 1.31 (0.50-3.80) 0.92 (0.31-2.71)  **WCRF Men: 0.90 (0.42-1.92) Women: 0.88 (0.37-2.05) |  |
| Ollberding et al., 2012 | Both | Cohort (MEC) | FFQ | Colorectal (incidence) | Red meat excluding processed red meat | Quintile 1 Quintile 2 Quintile 3 Quintile 4 Quintile 5 | 654/Not reported (n=33,143) 702/Not reported (n=33,143) 712/Not reported (n=33,144) 677/Not reported (n=33,144) 659/Not reported (n=33,143) | 1.00 (ref) 0.99 (0.89-1.11) 1.00 (0.90-1.12) 0.97 (0.87-1.09) 0.98 (0.87-1.10) | Combined |
| Bernstein et al., 2015 | Both | Cohort (NHS, HPFS) | FFQ | Colorectal (incidence) | Unprocessed red meat | per 1 serving/day | 2,731 (M: 996; W: 1,735)/ 134,497 (M: 47,389;  W: 87,108) | 0.99 (0.87-1.13) | Combined |
| Etemadi et al., 2018 | Both | Cohort (NIH-AARP, PLCO, AHS) | Diet history | Colorectal (incidence) | Unprocessed red meat | per 50 g/1,000 kcal/day | 6,640/407,270 | 1.34 (1.22-1.46) | Combined |
| Jones et al., 2019 | W | Cohort (IWHS) | FFQ | Colon (incidence) | Red meat | per 1-unit change in natural log-transformed value | 1,324/34,708 | 1.09 (1.02-1.16) | Combined, Men/Women |
|  |  |  |  | Rectal (incidence) | Red meat | per 1-unit change in natural log-transformed value | 325/34,708 | 1.08 (0.95-1.24) |  |
| Mehta et al., 2019 | W | Cohort (The Sister study) | FFQ and diet history | Colorectal (incidence) | Unprocessed red meat | per 50 g/day | 210/47,712 | 1.14 (0.88–1.48) | Combined, Men/Women |
| Pietinen et al., 1999 | M | Cohort (ATBC) | Diet history | Colorectal (incidence) | Beef, pork, lamb | Quartile 1  Quartile 2  Quartile 3  Quartile 4 | 55/Not reported 35/Not reported 50/Not reported 45/Not reported | 1.0 (ref) 0.6 (0.4-1.0) 0.9 (0.6-1.3) 0.8 (0.5-1.2) | Combined, Men/Women |
| Jarvinen et al., 2001 | Both | Cohort (FMCHES) | FFQ | Colorectal (incidence) | Red meat | Quartile 1  Quartile 2  Quartile 3  Quartile 4  **WCRF per 100 g/day | Not reported | Total: 1.00 (ref) 1.06 (0.67-2.01) 1.55 (0.88-2.73) 1.50 (0.77-2.94)  **WCRF 1.37 (0.92-2.06) | Combined |
| Larsson et al., 2005 | W | Cohort (SMC) | FFQ | Colorectal (incidence) | Beef and pork | <2.0 servings/week 2.0-<3.0 servings/week 3.0-<4.0 servings/week ≥4.0 servings/week  **WCRF per 100 g/day | Not reported | 1.00 (ref) 1.13 (0.95-1.36) 0.90 (0.70-1.17) 1.22 (0.98-1.53)  **WCRF 1.23 (0.90-1.67) | Combined, Men/Women |
| Vulcan et al., 2017 | M/W | Cohort (MDC) | Diet history | Colorectal (incidence) | Unprocessed red meat | Quintile 1 Quintile 2 Quintile 3 Quintile 4 Quintile 5 | Total: 141/85,679 147/85,967 150/85,640 146/85,624 144/86,014  Men: 52/24,888 65/29,689 71/31,959 79/34,519 89/43,588  Women: 89/60,792 82/56,278 79/53,682 67/51,105 55/42,426 | Total: 1.00 (ref) 1.00 (0.82-1.22) 0.88 (0.68-1.14) 0.98 (0.80-1.20) 1.08 (1.06-1.09)  Men: 1.00 (ref) 1.02 (0.71-1.47) 1.06 (0.74-1.52) 1.11 (0.78-1.58) 1.00 (0.70-1.43)  Women: 1.00 (ref) 0.98 (0.72-1.33) 1.03 (0.76-1.40) 0.96 (0.69-1.33) 1.03 (0.73-1.46) | Men/Women |
| Parr et al., 2013 | W | Cohort (NOWAC) | FFQ | Colorectal (incidence) | Red meat | <5 g/day 5-15 g/day 15-<25 g/day 25-<35 g/day ≥35 g/day | 99/118,590 287/347,171 190/227,003 57/91,454 33/45,201 | 1.00 (ref) 1.00 (0.80-1.26) 1.06 (0.83-1.36) 0.82 (0.58-1.14) 0.92 (0.61-1.39) | Men/Women |
| Gilsing et al., 2015 | Both | Cohort (NLCS-MIC) | FFQ | Colorectal (incidence) | Fresh red meat | per 50 g/day | 437/10,210 | 1.0 (0.90-1.11) | Combined |
| Diallo et al., 2018 | Both | Cohort (NutriNet-Sante) | 24-hour diet recall | Colorectal (incidence) | Red meat | Quintile 1 Quintile 2 Quintile 3 Quintile 4 Quintile 5 | 21/Not reported (n=12,313) 22/Not reported (n=12,235) 26/Not reported (n=12,282) 28/Not reported (n=12,206) 23/Not reported (n=12,320) | 1.00 (ref) 0.91 (0.49-1.67) 1.06 (0.59-1.91) 1.15 (0.65-2.06) 1.05 (0.57-1.93) | Combined |
| Knuppel et al., 2019 | Both | Cohort (UK Biobank) | FFQ | Colorectal (incidence) | Red meat | per 50 g/day | 3,194/475,023 | 1.22 (1.05-1.42) | Combined |
| Norat et al., 2005 | Both | Cohort (EPIC) | FFQ | Colorectal (incidence) | Red meat | per 100 g/day | 1,329 (M: 542, W: 787)/ 478,040 (M: 141,987, W: 336,052) | 1.21 (1.02-1.43) | Combined |
| English et al., 2004 | Both | Cohort (MCCS) | FFQ | Colorectal (incidence) | Fresh red meat | per 1 time/week | 451 (M: 225, W: 226)/ 37,112 | 1.03 (0.98-1.08) | Combined |
| Tiemersma et al., 2002 | M/W | Nested case-control (MP-CVDRF) | FFQ | Colorectal (incidence) | Fresh red meat | 0-3 times per week 3.1-4.5 times per week 5+ times per week | Total: 22/157 35/186 45/194  Men: 7/85 17/84 30/123  Women: 15/72 18/102 15/71 | Total: 1.0 (ref) 1.3 (0.7-2.3) 1.6 (0.9-2.9)  Men: 1.0 (ref) 2.7 (1.1-6.9) 2.7 (1.1-6.7)  Women:  1.0 (ref) 0.8 (0.4-1.8) 1.2 (0.5-2.8) | Combined, Men/Women |
| Kim et al., 2018 | M/W | Case-control (Multi-center hospital-based) | FFQ | Colorectal (incidence) | Red meat | <1 time/week 1-<3 timtes/week 3-<5 times/week ≥5 times/week | Total: 91/78 451/310 243/172 186/98 Men: 32/26 243/155 159/97 137/66 Women: 59/52 208/155 84/75 49/32 | Total: 1.00 (ref) 1.29 (0.91-1.84) 1.12 (0.76-1.64) 1.29 (0.83-2.01) Men: 1.00 (ref) 1.40 (0.78-2.51) 1.26 (0.68-2.33) 1.36 (0.70-2.64) Women: 1.00 (ref) 1.21 (0.77-1.88) 0.97 (0.58-1.63) 1.14 (0.59-2.20) | Combined, Men/Women |
| Kimura et al., 2007 | Both | Case-control (Population-based) | FFQ | Colorectal (incidence) | Beef/pork | Quintile 1 Quintile 2 Quintile 3 Quintile 4 Quintile 5 | 142/158 188/159 161/158 140/159 151/159 | 1.00 (ref) 1.35 (0.98-1.85) 1.28 (0.92-1.79) 1.03 (0.73-1.44) 1.13 (0.80-1.61) | Combined |
| Seow et al., 2002 | Both | Case-control (Population-based) | FFQ | Colorectal (incidence) | Red meat | Tertile 1  Tertile 2 Tertile 3 | 20/58 34/76 66/82 | 1.0 (ref) 1.1 (0.5-2.2) 2.2 (1.1-4.2) | Combined |
| Saliba et al., 2018 | Both | Case-control (Population-based) | FFQ | Colorectal (incidence) | Red meat | per 1 serving/week | 5,472  (Jews: 4615, Arabs:857)/ 10,026  (Jews: 8,615, Arabs: 1,411) | Jews: 1.05 (1.01-1.08)  Arabs: 0.94 (0.88-1.01) | Combined |
| **Processed meat and colorectal cancer** | | | | | | | | | |
| Islam et al., 2019* | M/W | Cohort  (JACC, MIYAGI, OHSAKI, JPHC-5y, TAKAYAMA) | Dietary questionnaire | Colorectal (incidence) | Ham, sausage | <1 time/week 1-2 times/week 3-4 times/week Almost every day | Men: 1,819/816,398 1,086/588,407 330/185,579 96/43,477  Women: 1,143/1,003,835 727/696,201 253/236,225 57/48,728 | Men: 1.00 (ref) 0.93 (0.85-1.01) 0.90 (0.79-1.02) 1.13 (0.91-1.41)  Women: 1.00 (ref) 1.06 (0.95-1.17) 1.11 (0.94-1.30) 1.33 (1.00-1.76) | Combined, Men/Women |
| Lin et al., 2004 | W | Cohort  (WHI-OS) | FFQ | Colorectal (incidence) | Processed meat | Quintile 1 Quintile 2 Quintile 3 Quintile 4 Quintile 5 | 51/Not reported 45/Not reported  42/Not reported  32/Not reported  32/Not reported | 1.00 (ref) 1.18 (0.79-1.77) 1.27 (0.84-1.91) 0.95 (0.60-1.49) 0.85 (0.53-1.35) | Combined, Women |
| Wei et al., 2004 | M/W | Cohort  (NHS, HPFS) | FFQ | Colon (incidence) | Processed meat | 0 servings per day <3 servings per month 1 serving per week 2 to 4 servings per week 5 or more servings per week  **WCRF per 50 g/day | Men: 122/Not reported 144/Not reported 89/Not reported 75/Not reported 37/Not reported  Women:  220/Not reported  169/Not reported  125/Not reported 110/Not reported  44/Not reported | Men: 1.00 (ref) 1.01 (0.79-1.30) 0.95 (0.71-1.27) 0.96 (0.71-1.30) 1.27 (0.87-1.85)  Women: 1.00 (ref) 0.96 (0.78-1.18) 1.10 (0.81-1.26) 1.21 (0.95-1.53) 1.32 (0.95-1.83)  **WCRF Men: 1.22 (0.87-1.77)  Women: 1.48 (1.10-2.00) | Men/Women |
|  |  |  |  | Rectal (incidence) | Processed meat | 0 servings per day <3 servings per month 1 serving per week 2 to 4 servings per week 5 or more servings per week  **WCRF per 50 g/day | Men: 35/Not reported  34/Not reported  37/Not reported 21/Not reported 8/Not reported  Women:  67/Not reported  61/Not reported  34/Not reported  33/Not reported 7/Not reported | Men: 1.00 (ref) 0.88 (0.54-1.44) 1.53 (0.94-2.52) 1.05 (0.59-1.86) 1.06 (0.48-2.33)  Women: 1.00 (ref) 1.13 (0.80-1.61) 0.89 (0.58-1.36) 1.18 (0.77-1.81) 0.73 (0.33-1.59)  **WCRF Men: 0.80 (0.40-1.60)  Women: 0.91 (0.47-1.75) | Men/Women |
| Chao et al., 2005 | M/W | Cohort (CPS-II) | FFQ | Colon (incidence) | Processed meat | Quintile 1 Quintile 2 Quintile 3 Quintile 4 Quintile 5 | Total: 153/Not reported 250/Not reported 321/Not reported 212/Not reported 261/Not reported  Men: 64/Not reported 125/Not reported 225/Not reported 108/Not reported 143/Not reported  Women: 89/Not reported 125/Not reported 96/Not reported 104/Not reported 118/Not reported | Total: 1.00 (ref) 0.90 (0.74-1.11) 1.01 (0.83-1.23) 1.02 (0.82-1.27) 1.13 (0.91-1.41)  Men: 1.00 (ref) 0.75 (0.55-1.02) 1.02 (0.76-1.36) 1.11 (0.80-1.54) 1.11 (0.80-1.54)  Women: 1.00 (ref) 1.11 (0.84-1.46) 0.95 (0.71-1.27) 0.94 (0.70-1.26) 1.16 (0.85-1.57) | Combined, Men/Women |
| Ollberding et al., 2012 | Both | Cohort (MEC) | FFQ | Colorectal (incidence) | Processed meat | Quintile 1 Quintile 2 Quintile 3 Quintile 4 Quintile 5 | 599/Not reported (n=33,143) 626/Not reported (n=33,143) 706/Not reported (n=33,143)  704/Not reported (n=33,143) 769/Not reported (n=33,143) | 1.00 (ref) 0.98 (0.87-1.09) 1.04 (0.93-1.16) 1.00 (0.90-1.13) 1.06 (0.94-1.19) | Combined |
| Jones et al., 2019 | W | Cohort (IWHS) | FFQ | Colon (incidence) | Processed meat | ≤0.74g/day 0.75-2.44g/day 2.45-4.59g/day >4.59g/day | 315/Not reported (n=11,565)  338/Not reported (n=6,028)  346/Not reported (n=8,794) 325/Not reported (n=8,321) | 1.00 (ref) 1.08 (0.92-1.26) 1.10 (0.94-1.28) 1.07 (0.91-1.25) | Combined, Women |
|  |  |  |  | Rectal (incidence) | Processed meat | ≤0.74g/day 0.75-2.44g/day 2.45-4.59g/day >4.59g/day | 85/Not reported (n=11,565)  77/Not reported (n=6,028)  84/Not reported (n=8,794)  79/Not reported (n=8,321) | 1.00 (ref) 0.92 (0.67-1.25) 0.99 (0.74-1.35) 0.97 (0.71-1.32) | Combined, Women |
| Flood et al., 2003 | W | Cohort (BCDDP) | FFQ | Colorectal (incidence) | Processed meat | Quintile 1 Quintile 2 Quintile 3 Quintile 4 Quintile 5  **WCRF per 50 g/day | Not reported | 1.00 (ref) 0.90 (0.68-1.18) 0.83 (0.63-1.11) 1.09 (0.84-1.43) 0.97 (0.73-1.28)  **WCRF 1.17 (0.76-1.81) | Combined, Women |
| Bernstein et al., 2015 | Both | Cohort (NHS, HPFS) | FFQ | Colorectal (incidence) | Processed red meat | per 1 serving/day | 2,731 (M: 996, W: 1,735)/ 134,497 (M: 47,389, W: 87,108) | 1.15 (1.01-1.32) | Combined |
| Etemadi et al., 2018 | Both | Cohort (NIH-AARP, PLCO, AHS) | AARP: FFQ PLCO: NCI-Diet History Questionnaire (DHQ)  AHS: NCI-DHQ | Colorectal (incidence) | Processed red meat | per 50 g/1,000 kcal/day | 6,640/407,270 | 1.30 (1.10-1.49) | Combined |
| Mehta et al., 2019 | W | Cohort (The Sister Study) | FFQ | Colorectal (incidence) | Processed meat | per 25g/day | 210/48,704 | 1.28 (0.98-1.67) | Combined, Women |
| Gaard et al., 1996 | M/W | Cohort (NNHSS) | FFQ | Colon (incidence) | Poached or fried sausages | <1 time/month 1-2 times/month 3-4 times/month ≥5 times/month | Men: 9/40,622 39/120,739 31/106,760 6/16,203  Women: 7/46,803 26/126,714 23/93,580 4/9,148 | Men: 1.00 (ref) 1.52 (0.74-3.14) 1.46 (0.69-3.06) 1.98 (0.70-5.58)  Women: 1.00 (ref) 1.45 (0.63-3.35) 1.85 (0.79-4.31) 3.50 (1.02-11.9) | Combined, Men/Women |
| Pietinen et al., 1999 | M | Cohort (ATBC) | Dietary questionnaire | Colorectal (incidence) | Processed meat | Quartile 1 Quartile 2  Quartile 3 Quartile 4 | 41/Not reported 58/Not reported 44/Not reported 42/Not reported | 1.0 (ref) 1.5 (1.0-2.2) 1.1 (0.7-1.8) 1.2 (0.7-1.8) | Combined, Men |
| English et al., 2004 | Both | Cohot (MCCS) | FFQ | Colorectal (incidence) | Processed meat | Quartile 1 Quartile 2  Quartile 3 Quartile 4 | 80/Not reported (n=7,509) 105/Not reported (n=7,688) 129/Not reported (n=12,460) 137/Not reported (n=9,455) | 1.0 (ref) 1.3 (1.0-1.7) 1.0 (0.8-1.4) 1.5 (1.1-2.0) | Combined |
| Larsson et al., 2005 | W | Cohort (SMC) | FFQ | Colorectal (incidence) | Processed meat | Quartile 1 Quartile 2  Quartile 3 Quartile 4  **WCRF per 50 g/day | Not reported | 1.00 (ref) 0.89 (0.72-1.90) 1.01 (0.82-1.24) 1.07 (0.85-1.33)  **WCRF 1.13 (0.85-1.51) | Combined, Women |
| Egeberg et al., 2013 | Both | Cohort (DCH) | FFQ | Colon (incidence) | Processed meat | Per 25 g/day increase | 644/53,988 (M: 25,832, W: 28,156) | 1.03 (0.94-1.13) | Combined |
|  |  |  |  | Rectal (incidence) | Processed meat | Per 25 g/day increase | 345/53,988 (M:25,832, W: 28,156) | 0.93 (0.81-1.07) | Combined |
| Parr et al., 2013 | W | Cohort (NOWAC) | FFQ | Colorectal (incidence) | Processed meat | <15 g/day  15-30 g/day 30-<45 g/day 45-<60 g/day ≥60 g/day | 141/153,865  205/247,618  153/212,704 81/117,351 94/99,734 | 1.00 (ref) 1.03 (0.83-1.28) 1.02 (0.80-1.29) 1.04 (0.78-1.38) 1.59 (1.19-2.12) | Combined, Women |
| Vulcan et al., 2017 | M/W | Cohort (MDC) | Dietary questionnaire | Colorectal (incidence) | Processed red meat | Quintile 1 Quintile 2 Quintile 3 Quintile 4 Quintile 5 | Total: 120/87,060 156/86,124 142/85,707 154/85,824 156/84,209  Men: 65/58,622 100/53,716 75/52,840  70/51,459 62/47,647  Women: 55/28,439 56/32,409 67/32,867  84/34,366 94/36,562 | Total: 1.00 (ref) 1.25 (0.98-1.59) 1.13 (0.88-1.45) 1.20 (0.94-1.53) 1.20 (0.94-1.53)  Men: 1.00 (ref) 1.62 (1.18-2.24) 1.25 (0.89-1.76) 1.18 (0.83-1.67) 1.13 (0.79-1.62)  Women: 1.00 (ref) 0.87 (0.60-1.26) 1.00 (0.70-1.43) 1.20 (0.85-1.69) 1.23 (0.87-1.73) | Combined, Men/Women |
| Diallo et al., 2018 | Both | Cohot (NutriNet-Sante) | 24-hour diet recall | Colorectal (incidence) | Processed meat | Quintile 1 Quintile 2 Quintile 3 Quintile 4 Quintile 5 | 27/Not reported (n=17,551) 18/Not reported (n=7,051) 25/Not reported (n=12,279)  31/Not reported (n=12,300)  19/Not reported (n=12,295) | 1.00 (ref) 1.42 (0.77-2.62) 1.19 (0.68-2.08) 1.56 (0.91-2.68) 1.10 (0.59-2.05) | Combined |
| Bradbury et al., 2019 | Both | Cohort (UK Biobank) | Dietary questionnaire | Colorectal (incidence) | Processed meat | 25g per day | 2,597/473,436 | 1.19 (1.03-1.38) | Combined |
| Balder et al., 2006 | M/W | Cohort (NLCS) | FFQ | Colorectal (incidence) | Total processed meat intake | 0g/day 0.1-9.9g/day 10-19.9g/day ≥20g/day | Men: 78/Not reported (n=279)  277/Not reported (n=961) 239/Not reported (n=849)  275/Not reported (n=936)   Women: 87/Not reported (n=383) 295/Not reported (n=1,282) 169/Not reported (n=708) 115/Not reported (n=508) | Men: 1.00 (ref) 1.02 (0.74-1.41) 0.98 (0.71-1.36) 1.18 (0.84-1.64)  Women: 1.00 (ref) 1.04 (0.78-1.39) 1.13 (0.82-1.55) 1.05 (0.74-1.48) | Combined, Men/Women |
| Spencer et al., 2010 | Both | Nested case-control (UK Dietary Cohort Consortium) | Food diaries | Colorectal (incidence) | Processed meat | per 50 g/day | 579/2,575 | 0.88 (0.68-1.15) | Combined |
| Kimura et al.. 2007 | Both | Case-control study (Population-based) | Dietary questionnaire | Colorectal (incidence) | Processed meat | Quintile 1 Quintile 2 Quintile 3 Quintile 4 Quintile 5 | 152/158 149/159 160/158  151/159 170/159 | 1.00 (ref) 1.03 (0.74-1.43) 1.09 (0.79-1.52) 1.07 (0.77-1.49) 1.15 (0.83-1.60) | Combined |
| Saliba et al., 2019 | Both | Case-control study (Population-based) | FFQ | Colorectal (incidence) | Processed meat | per 1 serving/week | 5,472 (Jews: 4,615, Arabs: 857)/ 10,026 (Jews: 8,615, Arabs: 1,411) | Jews: 1.02 (1.00-1.03) Arabs: 0.99 (0.91-1.07) | Combined |
| Kojima et al., 2004 | M/W | Cohot (JACC) | FFQ | Colon (mortality) | Ham and sausage | Low: 0-2 times per month Middle: 1-2 times per week High: 3-7 times per week | Men: 55/175,115  33/128,706  28/65,101   Women: 63/253,150  33/178,396 15/89,266 | Men 1.00 (ref) 0.89 (0.58-1.38) 1.44 (0.90-2.31)  Women 1.00 (ref) 0.94 (0.61-1.44) 0.94 (0.53-1.66) | Combined, Men/Women |
|  |  |  |  | Rectal (mortality) | Ham and sausage | Low: 0-2 times per month Middle: 1-2 times per week High: 3-7 times per week | Men: 48/175,115 29/128,706 16/65,101  Women: 19/253,150 9/178,396  9/89,266 | Men 1.00 (ref) 0.91 (0.57-1.45) 1.00 (0.56-1.78)  Women 1.00 (ref) 0.74 (0.33-1.65) 1.56 (0.69-3.53) | Combined, Men/Women |
| **Dietary fiber and colorectal cancer** | | | | | | | | | |
| Otani et al., 2006 | M/W | Cohort (JPHC-5y) | FFQ | Colorectal (incidence) | Dietary fiber | Quintile 1 Quintile 2 Quintile 3 Quintile 4 Quintile 5 | Men: 68/40,291 69/42,872  55/43,391  72/43,565  71/42,069  Women: 34/46,443  27/48,077  34/49,104 49/50,179  43/49,626 | Men: 1.00 (ref) 0.90 (0.63-1.3) 0.70 (0.47-1.1) 0.88 (0.58-1.3) 0.85 (0.53-1.4)  Women: 1.00 (ref) 0.61 (0.35-1.0) 0.62 (0.36-1.1) 0.77 (0.44-1.3) 0.58 (0.31-1.1) | Combined, Men/Women |
| Wakai et al., 2007 | M/W | Cohort (JACC) | FFQ | Colorectal (incidence) | Total dietary fiber | Quartile 1  Quartile 2  Quartile 3  Quartile 4 | Total: 97/78,849  114/81,220 102/83,145 130/84,059   Men: 51/30,631  76/31,855 54/32,224  77/32,187   Women: 46/48,219  38/49,366 48/50,921  53/51,872 | Total: 1.00 (ref) 0.96 (0.72-1.27) 0.72 (0.53-0.99) 0.73 (0.51-1.03)  Men: 1.00 (ref) 1.12 (0.77-1.62) 0.62 (0.40-0.96) 0.69 (0.43-1.11)  Women: 1.00 (ref) 0.73 (0.47-1.14) 0.84 (0.54-1.33) 0.75 (0.46-1.25) | Combined, Men/Women |
| Shin et al., 2006 | W | Cohort (SWHS) | FFQ | Colorectal (incidence) | Dietary fiber | Quintile 1 Quintile 2 Quintile 3 Quintile 4 Quintile 5  **WCRF per 10 g/day | Not reported | 1.00 (ref) 1.50 (1.00-2.20) 1.00 (0.60-1.60) 1.00 (0.60-1.60) 1.10 (0.60-1.80)  **WCRF 0.97 (0.61-1.53) | Combined, Women |
| Mai et al., 2003 | W | Cohort (BCDDP) | FFQ | Colorectal (incidence) | Total fibre | Quintile 1 Quintile 2 Quintile 3 Quintile 4 Quintile 5 | 99/Not reported 92/Not reported 72/Not reported 113/Not reported 111/Not reported | 1.00 (ref) 0.90 (0.67–1.19) 0.67 (0.49–0.91) 1.00 (0.76–1.33) 0.94 (0.70–1.26) | Women |
| McCullough et al., 2003 | M/W | Cohort (CPS-II) | FFQ | Colon (incidence) | Dietary fiber | Quintile 1 Quintile 2 Quintile 3 Quintile 4 Quintile 5 | Men: 81/Not reported 49/Not reported 49/Not reported 59/Not reported  60/Not reported   Women: 40/Not reported 51/Not reported 33/Not reported  55/Not reported 31/Not reported | Men: 1.00 (ref) 0.63 (0.44-0.92) 0.64 (0.43-0.96) 0.84 (0.55-1.28) 1.01 (0.62-1.65)  Women: 1.00 (ref) 1.41 (0.90-2.19) 1.04 (0.62-1.75) 1.79 (1.07-2.99) 1.09 (0.58-2.05) | Men/Women |
| Lin et al., 2005 | W | Cohort (WHI-OS) | FFQ | Colorectal (incidence) | Total fiber | Quintile 1 Quintile 2 Quintile 3 Quintile 4 Quintile 5 | 43/Not reported 44/Not reported 33/Not reported  50/Not reported  53/Not reported | 1.00 (ref) 0.90 (0.59–1.38) 0.62 (0.39–0.98) 0.84 (0.54–1.31) 0.75 (0.47–1.18) | Women |
| McCarl et al., 2006 | W | Cohort (IWHS) | FFQ | Colorectal (incidence) | Fiber | Quintile 1 Quintile 2 Quintile 3 Quintile 4 Quintile 5 | 205/92,808  185/93,590 200/95,353 200/94,507 164/95,251 | 1.00 (ref) 0.89 (0.73-1.09) 0.93 (0.77-1.13) 0.93 (0.77-1.14) 0.75 (0.61-0.92) | Women |
| Schatzkin et al., 2007 | M/W | Cohort (NIH-AARP) | FFQ | Colorectal (incidence) | Dietary fiber intake | Quintile 1 Quintile 2 Quintile 3 Quintile 4 Quintile 5 | Total: 704/423,602  617/425,196  585/425,675  531/426,553  537/427,501  Men: 470/251,397  425/252,152  413/251,938 353/252,355 388/252,626   Women: 205/172,759 192/173,683 168/173,795 164/173,696  196/174,126 | Total: 1.00 (ref) 0.92 (0.82-1.03) 0.93 (0.82-1.06) 0.90 (0.78-1.04) 0.99 (0.85-1.15)  Men: 1.00 (ref) 0.94 (0.82-1.09) 0.98 (0.84-1.14) 0.89 (0.75-1.05) 1.06 (0.88-1.28)  Women: 1.00 (ref) 0.94 (0.76-1.16) 0.86 (0.68-1.08) 0.88 (0.69-1.13) 1.10 (0.84-1.43) | Combined, Men/Women |
| Kunzmann et al., 2015 | Both | Cohort (PLCO) | FFQ | Colorectal (incidence) | Total fiber | Tertile 1 Tertile 2  Tertile 3 | 262/211,601 246/215,671 225/217,035 | 1.00 (ref) 0.94 (0.79-1.13) 0.85 (0.70-1.03) | Combined |
| Navarro et al., 2016 | W | Cohort (WHI-OS) | FFQ | Colorectal (incidence) | Total fiber | Quintile 1 Quintile 2 Quintile 3 Quintile 4 Quintile 5 | 420/302,828 426/312,546 354/316,244 372/318,893 380/318,844 | 1.00 (ref) 1.00 (0.87-1.15) 0.83 (0.71-0.97) 0.87 (0.74-1.03) 0.90 (0.73-1.10) | Combined, Women |
| Park et al., 2016 | M/W | Cohort (MEC) | FFQ | Colorectal (incidence) | Dietary fiber | Quintile 1 Quintile 2 Quintile 3 Quintile 4 Quintile 5 | Men: 703/Not reported (n=23,104)  501/Not reported (n=19,100)  420/Not reported (n=16,750) 354/Not reported (n=14,618) 260/Not reported (n=12,333)  Women: 273/Not reported (n=13,919) 341/Not reported (n=18,351) 406/Not reported (n=20,864) 462/Not reported (n=23,171) 455/Not reported (n=25,464) | Men: 1.00 (ref) 0.86 (0.76–0.97) 0.83 (0.72–0.95) 0.82 (0.70–0.96) 0.73 (0.61–0.89)  Women: 1.00 (ref) 0.88 (0.75–1.04)  0.88 (0.75–1.04)  0.87 (0.73–1.03)  0.76 (0.62–0.91) | Combined, Men/Women |
| He et al., 2019 | M/W | Cohort (NHS, HPFS) | FFQ | Colorectal (incidence) | Total fiber | per 5 g/day | 3,178 (M: 1,276, W: 1,902) /138,793 (M: 47,924, W: 90,869) | Men: 0.98 (0.92-1.04)  Women: 1.02 (0.95–1.09) | Men/Women |
| Gaard et al., 1996 | M/W | Cohort (NNHSS) | FFQ | Colon (incidence) | Fiber | Quartile 1  Quartile 2  Quartile 3  Quartile 4 | Men: 22/74,009  20/73,293 22/70,870 19/70,745  Women: 9/72,209 13/70,400 20/70,522 17/68,794 | Men: 1.00 (ref) 0.85 (0.43-1.37) 0.93 (0.30-1.09) 0.82 (0.46-1.46)  Women: 1.00 (ref) 1.73 (0.73-4.13) 2.42 (1.06-5.51) 2.10 (0.90-4.87) | Combined, Men/Women |
| Pietinen et al., 1999 | M | Cohort (ATBC) | Dietary questionnaire | Colorectal (incidence) | Dietary fiber | Quartile 1  Quartile 2  Quartile 3  Quartile 4 | 44/Not reported 48/Not reported 47/Not reported 46/Not reported | 1.0 (ref) 1.0 (0.7-1.6) 1.0 (0.7-1.5) 1.0 (0.6-1.5) | Men |
| Terry et al., 2001 | W | Cohort (SMC) | FFQ | Colorectal (incidence) | Cereal fiber | Quartile 1  Quartile 2  Quartile 3  Quartile 4 | 104/146,602  87/146,978 144/147,280 125/147,410 | 1.00 (ref) 0.80 (0.60-1.07) 1.20 (0.93-1.56) 0.91 (0.69-1.20) | Women |
| Sanjoaquin et al., 2004 | Both | Cohort (OVS) | FFQ | Colorectal (incidence) | Total dietary fibre | Tertile 1  Tertile 2 Tertile 3 | 20/Not reported  24/Not reported 19/Not reported | 1.00 (ref) 1.07 (0.59-1.95) 0.82 (0.43-1.56) | Combined |
| Bradbury et al., 2019 | Both | Cohort (UK Biobank) | FFQ | Colorectal (incidence) | Fibre | Quintile 1 Quintile 2 Quintile 3 Quintile 4 Quintile 5 | 487/Not reported (n=91,213)  501/Not reported (n=90,766)  489/Not reported (n=90,396) 481/Not reported (n=90,481)  499/Not reported (n=90,288) | 1.00 (ref) 0.99 (0.88-1.13) 0.96 (0.84-1.09) 0.93 (0.81-1.06) 0.94 (0.83-1.07) | Combined |
| Park et al., 2005* | Both | Cohort (Pooling Project) | study specific FFQ | Colorectal (incidence) | Dietary fiber | 10-<15 g/day <10 g/day 15-<20 g/day 20-<25 g/day 25-<30 g/day ≥30 g/day | 609/513,317 1,681/1,591,322  2,263/1,870,758 1,740/1,183,334 1,001/514,142 785/313,572 | 1.00 (ref) 1.18 (1.05-1.31) 1.02 (0.95-1.10) 1.01 (0.92-1.10) 0.99 (0.87-1.12) 1.00 (0.85-1.17) | Combined |
| Murphy et al., 2012 | M/W | Cohort (EPIC) | country/centre specific dietary questionnaires | Colorectal (incidence) | Total fiber | per 10 g/day | 4,517 (M: 1,904, W: 2,613)/477,312 (M: 142,250, W: 335,062) | Total: 0.87 (0.79–0.96)  Men: 0.91 (0.83-1.01)  Women: 0.89 (0.80-0.97) | Combined, Men/Women |
| Andersen et al., 2019 | Both | Cohort (DCH) | FFQ | Colorectal (incidence) | Dietary fiber | per 10 g/day | 1,038 (M: 576, W:462)/57,053 | 0.83 (0.65–1.08) | Combined |
| Uchida et al., 2010 | Both | Case-control (Population-based) | Dietary history interview | Colorectal (incidence) | Dietary fiber | Quintile 1 Quintile 2 Quintile 3 Quintile 4 Quintile 5 | 175/163 152/163 178/163 153/163 158/163 | 1.00 (ref) 0.87 (0.62–1.21) 0.95 (0.67–1.34) 0.81 (0.56–1.18) 0.87 (0.58–1.31) | Combined |
| Huang et al., 2018* | M/W | Case-control (Population-based) | FFQ | Colorectal (incidence) | Total fibre | Quartile 1  Quartile 2  Quartile 3  Quartile 4 | Total: 779/506 491/508 357/507 317/506  Men: 473/268 273/269 171/268 162/268  Women: 306/238 218/239 186/239  155/238 | Total: 1.00 (ref) 0.65 (0.55-0.79) 0.50 (0.42-0.61) 0.47 (0.39-0.58)  Men: 1.00 (ref) 0.59 (0.46-0.75) 0.35 (0.27-0.46) 0.56 (0.27-0.47)  Women: 1.00 (ref) 0.80 (0.60-1.08) 0.81 (0.59-1.09) 0.76 (0.55-1.05) | Combined, Men/Women |
| Song et al., 2018 | Both | Cohort (NHS, HPFS) | FFQ | Colorectal (mortality) | Total fiber | Quartile 1  Quartile 2  Quartile 3  Quartile 4 | 56/3,335 40/3,614 40/3,475 38/3,786 | 1.00 (ref) 0.72 (0.50-1.03) 0.48 (0.32-0.71) 0.54 (0.35-0.85) | Combined |
| Jansen et al., 1999 | M | Cohort (SCS) | Dietary record | Colorectal (mortality) | Dietary fiber | per 10% of mean intake (3.32 g/day) | 162/12,763 | 0.89 (0.80-0.97) | Combined, Men |
| Ward et al., 2016* | Both | Cohort (EPIC) | Country-specific FFQ | Colorectal (mortality) | Fibre | Quartile 1  Quartile 2  Quartile 3  Quartile 4 | 259/Not reported  249/Not reported  265/Not reported 235/Not reported | 1.00 (ref) 0.95 (0.79-1.15) 1.04 (0.85-1.27) 0.90 (0.69-1.17) | Combined |
| **Salted vegetables and stomach cancer** | | | | | | | | | |
| Kato et al., 1992 | Both | Cohort (HERPACC) | FFQ | Stomach (incidence) | Pickles | ≤1-2 times per month 2-3 times per week Daily | 5/3017.5 15/7330.5 21/6479.3 | 1.00 (ref) 1.26 (0.46-3.48) 1.77 (0.67-4.70) | Combined |
| Sauvaget et al., 2005* | Both | Cohort (LSS) | FFQ | Stomach (incidence) | Pickles | <2 times per week 2-4 times per week 5+ times per week | 410/150,521 233/107,738 627/226,315 | 1.00 (ref) 0.91 (0.77-1.07) 1.11 (0.98-1.26) | Combined |
| Takachi et al., 2010* | Both | Cohort (JPHC) | FFQ | Stomach (incidence) | Pickled vegetables | Quintile 1 Quintile 2 Quintile 3 Quintile 4 Quintile 5 | 95/Not reported  175/Not reported 168/Not reported  212/Not reported 217/Not reported | 1.00 (ref) 1.91 (1.47-2.48) 1.70 (1.30-2.22) 2.14 (1.65-2.77) 2.24 (1.71-2.93) | Combined |
| Nomura et al., 1990 | M | Cohort (HHP) | FFQ | Stomach (incidence) | Pickles | ≤1 serving per week 2-4 servings per week ≥5 servings per week | 54/Not reported (n=3,097) 31/Not reported (n=1,950) 65/Not reported (n=2,941) | 1.00 (ref) 0.90 (0.60-1.50) 1.20 (0.80-1.70) | Combined, Men |
| Galanis et al, 1998 | M/W | Cohort (Hawaii-Japan DOH Survey) | FFQ | Stomach (incidence) | Pickled vegetables | None 1-6 times per week ≥7 times per week | Total: 29/Not reported (n=3,993) 42/Not reported (n=4,621)  37/Not reported (n=3,293)  Men: 19/Not reported (n=1,828) 24/Not reported (n=2,208) 21/Not reported (n=1,574)  Women: 10/Not reported (n=2,165) 18/Not reported (n=2,413)  16/Not reported (n=1,719) | Total: 1.00 (ref) 1.30 (0.80-2.20) 1.10 (0.70-1.80)  Men: 1.00 (ref) 1.20 (0.60-2.10) 0.90 (0.50-1.80)  Women: 1.00 (ref) 1.70 (0.80-3.70) 1.40 (0.60-3.10) | Combined, Men/Women |
| Machida-Montani et al., 2004 | Both | Case-control (Multi-center hospital-based) | FFQ | Stomach non-cardia (incidence) | Pickled vegetables | Tertile 1  Tertile 2  Tertile 3 | 52/79 33/78 37/78 | 1.0 (ref) 0.6 (0.3-1.2) 0.6 (0.3-1.3) | Combined |
| Cai et al., 2003 | Both | Case-control (Multi-center hospital-based) | FFQ | Stomach cardia (incidence) | Pickled vegetables intake | <1 time per month <3 times per week ≥3 times per week | 61/88  63/76 67/58 | 1.00 (ref) 1.335 (0.804-2.214) 1.762 (1.044-2.974) | Combined |
|  |  |  |  | Stomach non-cardia (incidence) | Pickled vegetables intake | <1 time per month <3 times per week ≥3 times per week | 69/88  64/76  57/58 | 1.00 (ref) 1.139 (0.701-1.852) 1.269 (0.766-2.103) | Combined |
| Sun et al., 2013 | Both | Case-control (Population-based) | FFQ | Stomach cardia (incidence) | Pickled food taking | Never 1-2 times per week ≥2 times per week | Not reported | Total: 1.00 (ref) 1.09 (0.80-1.48) 1.46 (1.02-2.09) | Combined |
| Hamada et al., 2002 | Both | Case-control (Multi-center hospital-based) | Dietary questionnaire | Stomach (incidence) | Pickled vegetables, Japanese style | <1 day per week 1-2 days per week 3-4 days per week Daily | 40/75 17/43  13/17  26/57 | 1.0 (ref) 0.7 (0.4-1.4) 1.4 (0.6-3.3) 1.0 (0.5-1.9) | Combined |
| Pakseresht et al., 2011* | Both | Case-control (Population-based) | FFQ | Stomach (incidence) | Pickles | per 10g/day | 286 (M:210, W:76)/590 (M: 427, W: 163) | 0.83 (0.74-0.93) | Combined |
| Kato et al., 1992 | Both | Cohort (Higashi-Kamo Cohort) | Dietary questionnaire | Stomach (mortality) | Pickles | ≤1-2 times per week 3-4 times per week Daily | 11/8,216.1 5/7,920.0 33/35,608.7 | 1.00 (ref) 0.51 (0.18-1.48) 0.75 (0.38-1.49) | Combined |
| Ngoan et al., 2002* | M/W | Cohort (FPC) | FFQ | Stomach (mortality) | Pickled food | Low: ≤2-4 times/week Medium: 1 times/day High: ≥2 times/day | Total: 15/Not reported (n=3,077) 29/Not reported (n=3,393) 18/Not reported (n=1,319)  Men: 10/Not reported (n=1,448)  24/Not reported (n=1,523) 14/Not reported (n=479)   Women: 5/Not reported (n=1,629)  5/Not reported (n=1,870) 4/Not reported (n=840) | Total: 1.00 (ref) 1.30 (0.70-2.50) 1.50 (0.70-3.20)  Men: 1.00 (ref) 1.90 (1.00-4.10) 2.60 (1.10-5.80)  Women: 1.00 (ref) 0.70 (0.20-2.60) 1.10 (0.30-4.30) | Combined, Men/Women |
| Iso and Kubota, 2007 | M/W | Cohort (JACC) | FFQ | Stomach (mortality) | Pickles | <3 times per week 3-4 times per week ≥5 times per week | Men: 162/128,779 90/88,665  457/322,125  Women: 70/166,459 41/109,129 234/489,208 | Men: 1.00 (ref) 0.87 (0.67-1.14) 1.07 (0.89-1.30) Women: 1.00 (ref) 0.96 (0.65-1.43) 1.08 (0.82-1.42) | Combined, Men/Women |
| **Salted fish and stomach cancer** | | | | | | | | | |
| Takachi et al., 2010 | Both | Cohort (JPHC) | FFQ | Stomach (incidence) | Dried and salted fish | Quintile 1 Quintile 2 Quintile 3 Quintile 4 Quintile 5 | 129/Not reported 195/Not reported  199/Not reported  173/Not reported 171/Not reported | 1.00 (ref) 1.56 (1.22-1.98) 1.57 (1.24-2.00) 1.48 (1.15-1.89) 1.46 (1.14-1.88) | Combined |
| Cai et al., 2003 | Both | Case-control (Multi-center hospital-based) | FFQ | Stomach cardia (incidence) | Salty fish intake | <1 time per month <3 times per week ≥3 times per week | 139/170  41/49  11/3 | 1.00 (ref) 1.019 (0.615-1.690) 5.518 (1.367-19.461) | Combined |
|  |  |  |  | Stomach non-cardia (incidence) | Salty fish intake | <1 time per month <3 times per week ≥3 times per week | 134/170  41/49 15/3 | 1.00 (ref) 1.058 (0.646-1.730) 5.706 (1.588-20.490) | Combined |
| Pakseresht et al., 2011 | Both | Case-control (Population-based) | FFQ | Stomach (incidence) | Salted fish | per 10g/day | 286 (M:210, W:76)/ 590(M: 427, W: 163) | 0.86 (0.63-1.18) | Combined |
| Ngoan et al., 2002 | M/W | Cohort (FPC) | FFQ | Stomach (mortality) | Processed fish | Low: ≤2-4 times/week Medium: 1 times/day High: ≥2 times/day | Total: 18/Not reported (n=2,943) 29/Not reported (n=3,975) 11/Not reported (n=902)  Men: 13/Not reported (n=1,324) 25/Not reported (n=1,737) 9/Not reported (n=423)  Women: 5/Not reported (n=1,620)  4/Not reported (n=2,238)  2/Not reported (n=479) | Total: 1.0 (ref) 1.1 (0.6-2.2) 1.1 (0.5-2.8)  Men: 1.0 (ref) 1.6 (0.8-3.1) 2.1 (0.9-4.9)  Women: 1.0 (ref) 0.7 (0.2-2.5) 1.4 (0.3-7.3) | Combined, Men/Women |
| Iso and Kubota, 2007 | M/W | Cohort (JACC) | FFQ | Stomach (mortality) | Dried or salted fish | <1 time per week 1-2 times per week ≥3-4 times per week | Men: 330/247,290 194/164,505  162/121,069  Women: 174/342,371 81/230,152 78/175,602 | Men: 1.00 (ref) 1.06 (0.85-1.31) 1.12 (0.89-1.40)  Women: 1.00 (ref) 0.74 (0.55-1.01) 0.92 (0.67-1.26) | Combined, Men/Women |
| **Salted fish and nasopharyngeal cancer** | | | | | | | | | |
| Zheng et al., 1994* | Both | Case-control  (Population-based) | Dietary questionnaire | Nasopharyngeal (incidence) | Salted fish | Never and yearly Monthly Weekly and daily | 35/102 49/74 67/19 | 1.0 (ref) 1.9 (1.1-3.4) 12.9 (5.2-20.6) | Combined |
| Yuan et al., 2000 | M/W | Case-control  (Population-based) | Dietary questionnaire | Nasopharyngeal (incidence) | Salted fish | Total: Less than monthly Monthly Weekly | Total: 802/913 113/108 20/11  Men: 578/620 80/73 10/6 Women: 224/293 33/35 10/5 | Total: 1.00 (ref) 1.17 (0.88–1.57)  1.82 (0.86–3.88)  Men: 1.00 (ref) 1.18 (0.83-1.67) 1.45 (0.51-4.08) Women: 1.00 (ref) 1.18 (0.70-2.00) 2.29 (0.76-6.93) | Combined, Men/Women |
| Ren et al., 2010 | Both | Case-control  (Hospital-based) | Dietary questionnaire | Nasopharyngeal (incidence) | Salt-preserved fish | Never or rarely ≥1 time per month ≥4 times per week | 990/1680 313/198 531/373 | 1.00 (ref) 2.68 (2.21-3.26) 2.42 (2.07-2.82) | Combined |
| Xu et al., 2012* | M | Case-control  (Hospital-based) | Dietary questionnaire | Nasopharyngeal (incidence) | Salted fish | Less than monthly Monthly Weekly or more | 1,046/1,398 150/80 115/85 | 1.00 (ref) 2.50 (1.87-3.33) 1.74 (1.29-2.35) | Men/Women |
| Barrett et al., 2019 | Both | Case-control  (Population-based) | FFQ | Nasopharyngeal (incidence) | Salted fish | Men: 0 g/day >0-≤0.41 g/day >0.41-≤1.64 g/day >1.64 g/day; W Women: 0 g/day >0-≤0.34 g/day >0.34-≤1.36 g/day >1.36 g/day | Total: 846/797 489/585 489/582 678/596 | Total: 0.81 (0.68-0.96) 0.73 (0.62-0.87) 0.93 (0.78-1.10) | Combined |
| Sriamporn et al., 1992 | Both | Case-control  (Multi-center hospital-based) | Dietary questionnaire | Nasopharyngeal (incidence) | Salted fish | Only fresh-water fish Less than once a week At least once a week | 69/74 16/22 35/24 | 1.00 (ref) 1.5 (0.6-3.5) 2.5 (1.2-5.2) | Combined |
| Fachiroh et al., 2012* | Both | Case-control  (Multi-center hospital-based) | Dietary questionnaire | Nasopharyngeal (incidence) | Salted fish | Never to rarely  Less than weekly Weekly or more | 340/601 220/313 121/164 | 1.00 (ref) 0.99 (0.77–1.27) 0.92 (0.68–1.25) | Combined |
| Yong et al., 2017 | Both | Case-control  (Population-based) | Dietary questionnaire | Nasopharyngeal (incidence) | Salted fish | Never/rarely Monthly Weekly | 251/264 32/20 7/3 | 1.00 (ref) 1.67 (0.93-2.99) 2.33 (0.60-9.02) | Combined |
| Yang et al., 2005 | Both | Case-control  (Population-based) | Dietary questionnaire | Nasopharyngeal (incidence) | Salted fish | Never <1/week ≥1/week | 342/1528 13/72 11/36 | 1.00 (ref) 0.65 (0.31-1.35) 1.78 (0.82-3.89) | Combined |
| **Non-starch vegetable and fruit (aggregated) and aerodigestive and some other cancer (colorectal, gastric, and lung cancer)** | | | | | | | | | |
| Boeing et al., 2006 | M/W | Cohort (EPIC) | Country/centre specific dietary questionnaires | Upper aerodigestive tract (incidence) | Total vegetables and fruit | per 80 g/day | 352/ 345,904(M: 130,633; W: 215,271) | Total: 0.91 (0.83-1.00)  Men: 0.88 (0.79-0.98)  Women: 0.96 (0.79-1.15) | Combined , Men/Women |
| Wie et al., 2014 | Both | Cohort (CSECK) | 3d food record | Colorectal (incidence) | Vegetables and fruit | per 100 g/day | 53 (M: 23, W: 30)/8,024 | 1.00 (0.88, 1.14) | Combined, Men/Women |
| Lee et al., 2009 | W | Cohort (SWHS) | FFQ | Colorectal (incidence) | Vegetables and fruit | Quartile 1  Quartile 2  Quartile 3  Quartile 4 | 114/133,940 93/135,103 96/135,817 91/135,295 | 1.0 (ref) 1.0 (0.8-1.3) 1.1 (0.9-1.5) 1.2 (0.9-1.6) | Combined, Men/Women |
| Vogtmann et al., 2013 | M | Cohort (SMHS) | FFQ | Colorectal (incidence) | Total vegetables and fruit | Quintile 1 Quintile 2 Quintile 3 Quintile 4 Quintile 5 | 88/Not reported 73/Not reported 85/Not reported 79/Not reported 73/Not reported | 1.00 (ref) 0.82 (0.60-1.12) 0.93 (0.68-1.26) 0.85 (0.61-1.17) 0.71 (0.50-1.01) | Combined, Men/Women |
| Lin et al., 2005 | W | Cohort  (WHS) | FFQ | Colorectal (incidence) | Vegetables and fruit | Quintile 1 Quintile 2 Quintile 3 Quintile 4 Quintile 5 | 42/Not reported 43/Not reported 44/Not reported 42/Not reported 52/Not reported | 1.00 (ref) 0.99 (0.64-1.53) 0.95 (0.60-1.50) 0.84 (0.51-1.37) 0.96 (0.58-1.62) | Combined, Men/Women |
| McCarl et al., 2006 | W | Cohort  (IWHS) | FFQ | Colorectal (incidence) | Vegetables and fruit | ≤27.4 servings/week 27.5-<36.4 servings/week 36.5-<45 servings/week 45.14-<58 servings/week ≥58.01 servings/week | 196/93,655 201/94,049 201/95,574 175/95,528 181/92,702 | 1.00 (ref) 1.01 (0.83-1.23) 0.98 (0.81-1.20) 0.85 (0.69-1.04) 0.90 (0.73-1.10) | Combined, Men/Women |
| Park et al., 2007 | M/W | Cohort  (NIH-AARP) | FFQ | Colorectal (incidence) | Total vegetables and fruit | Quintile 1 Quintile 2 Quintile 3 Quintile 4 Quintile 5  **WCRF per 100 g/day | Men: 502/Not reported 393/Not reported 393/Not reported 366/Not reported 394/Not reported  Women: 188/Not reported 198/Not reported 165/Not reported 191/Not reported 182/Not reported | Men: 1.00 (ref) 0.82 (0.71-0.93) 0.84 (0.70-0.93) 0.81 (0.70-0.93) 0.91 (0.78-1.05)  Women: 1.00 (ref) 1.09 (0.89-1.34) 0.93 (0.75-1.15) 1.11 (0.90-1.38) 1.08 (0.86-1.35)  **WCRF Men: 0.99 (0.97-1.01) Women: 1.01 (0.98-1.05) | Combined |
| Nomura et al., 2008 | M/W | Cohort  (MEC) | FFQ | Colorectal (incidence) | Vegetables and fruit | Quintile 1 Quintile 2 Quintile 3 Quintile 4 Quintile 5   **WCRF per 100 g/day | Not reported | Men: 1.00 (ref) 0.88 (0.72-1.06) 0.84 (0.69-1.02) 0.84 (0.69-1.03) 0.74 (0.59-0.93)  Women: 1.00 (ref) 1.04 (0.82-1.30) 1.04 (0.83-1.31) 0.94 (0.74-1.20) 1.04 (0.81-1.33)  **WCRF Men: 0.97 (0.94-0.99) Women: 1.00 (0.97-1.03) | Combined |
| Makarem et al., 2015 | Both | Cohort  (FHS-Offspring) | FFQ | Colorectal (incidence) | Vegetables and fruit | per 100 g/day | 63/2,983 | 0.96 (0.48-1.94) | Combined |
| Kunzmann et al., 2016 | M/W | Cohort  (PLCO) | FFQ | Colorectal (incidence) | Vegetables and fruit (excluding juice) | Tertile 1 Tertile 2 Tertile 3 | Total: 268/211,747 242/216,254 223/216,306  Men: 196/177,711 152/112,441 81/71,026  Women: 72/64,036 90/103,813 142/145,280 | Total: 1.00 (ref) 0.90 (0.75-1.08) 0.84 (0.69-1.02)  Men: 1.00 (ref) 0.99 (0.80-1.23) 0.80 (0.61-1.05)  Women: 1.00 (ref) 0.74 (0.54-1.02) 0.80 (0.59-1.07) | Combined, Men/Women |
| Voorrips et al., 2000 | M/W | Cohort (NLCS) | FFQ | Colon (incidence) | Total vegetables and fruit | Quintile 1 Quintile 2 Quintile 3 Quintile 4 Quintile 5 | Men: 62/1,739 55/1,694 66/1,777 65/1,715 64/1,748  Women: 71/1,794 42/1,714 54/1,765 54/1,770 45/1,762 | Men: 1.00 (ref) 0.85 (0.57-1.28) 1.00 (0.67-1.47) 1.00 (0.68-1.48) 0.95 (0.64-1.41)  Women: 1.00 (ref) 0.63 (0.41-0.97) 0.77 (0.52-1.15) 0.77 (0.51-1.14) 0.66 (0.44-1.01) | Combined, Men/Women |
| , |  |  |  | Rectal (incidence) | Total vegetables and fruit | Quintile 1 Quintile 2 Quintile 3 Quintile 4 Quintile 5 | Men: 47/1,743 34/1,694 31/1,779 43/1,716 44/1,760  Women: 20/1,797 24/1,706 26/1,765 22/1,771 23/1,766 | Men: 1.00 (ref) 0.72 (0.44-1.15) 0.63 (0.39-1.02) 0.90 (0.57-1.41) 0.88 (0.56-1.37)  Women: 1.00 (ref) 1.27 (0.69-2.35) 1.33 (0.73-2.43) 1.08 (0.58-2.03) 1.17 (0.63-2.17) |  |
| van Duijnhoven et al., 2009 | Both | Cohort  (EPIC) | Country/centre specific dietary questionnaires | Colorectal (incidence) | Vegetables and fruit | per 100g/day | 2,819/452,755 | 0.98 (0.97-1.00) | Combined |
| Luo et al., 2015* | M/W | Case-control (Hospital and population-based) | FFQ | Colorectal (incidence) | Total vegetables and fruit | Quartile 1  Quartile 2 Quartile 3  Quartile 4 | 380/263 280/266 243/265  154/263 | 1.00 (ref) 0.68 (0.53-0.87) 0.58 (0.44-0.74) 0.37 (0.28-0.49) | Combined, Men/Women |
| Yamaji et al., 2008 | M | Cohort  (JPHC I, JPHC II) | FFQ | Esophageal squamous cell carcinoma (incidence) | Total vegetables and fruit | per 100g/day | 141/38,790 | 0.89 (0.79-0.99) | Combined, Men/Women |
| Freedman et al., 2007* | Both | Cohort  (NIH-AARP) | FFQ | Esophageal squamous cell carcinoma (incidence) | Total vegetables and fruit | per serving/1,000 kcal | 103/ 490,802 (M: 292,898, W: 197,904) | 0.78 (0.67-0.91) | Combined |
|  |  |  |  | Esophageal adenocarcinoma (incidence) | Total vegetables and fruit | per serving/1,000 kcal | 213/ 490,802 (M: 292,898, W: 197,904) | 0.98 (0.90–1.08) | Combined |
| Maasland et al., 2015 | Both | Cohort study (NLCS) | FFQ | Head and neck (incidence) | Total vegetables and fruit | per 25 g/day | 415/65,980 | 0.97 (0.95–0.99) | Combined |
| Wie et al., 2014 | Both | Cohort (CSECK) | Dietary records | Lung (incidence) | Vegetables and fruit | per 100 g/day | 36/8,024 | 0.82 (0.57, 1.17) | Combined |
| Wakai et al., 2015* | M/W | Cohort  (JPHC I , JPHC II,JACC, MIYAGI) | FFQ | Lung (incidence) | Vegetables and fruit | Quintile 1 Quintile 2 Quintile 3 Quintile 4 Quintile 5 | Men: 298/Not reported 258/Not reported 263/Not reported 289/Not reported 277/Not reported  Women: 91/Not reported 67/Not reported 69/Not reported 91/Not reported 83/Not reported | Men: 1.00 (ref) 1.07 (0.90–1.28) 0.95 (0.79–1.14) 1.12 (0.94–1.33) 1.07 (0.90–1.28)  Women: 1.00 (ref) 1.11 (0.82–1.49) 0.94 (0.69–1.28) 1.07 (0.80–1.45) 1.04 (0.77–1.41) | Combined, Men/Women |
| Slatore et al., 2008 | Both | Cohort  (VITAL) | FFQ | Lung (incidence) | Vegetables and fruit | Quartile 1  Quartile 2  Quartile 3 Quartile 4 | 134/Not reported (n=17,250) 130/Not reported (n=17,486)  100/Not reported (n=17,383)  84/Not reported (n=17,489) | 1.00 (ref) 1.08 (0.85–1.38) 0.91 (0.70–1.19) 0.90 (0.68–1.19) | Combined |
| Wright et al, 2008 | M/W | Cohort  (NIH-AARP) | FFQ | Lung (incidence) | Total vegetables and fruit | Quintile 1 Quintile 2 Quintile 3 Quintile 4 Quintile 5 | Men: 1,212/Not reported (n=56,257)  809/Not reported (n=56,258)  681/Not reported (n=56,258)  600/Not reported (n=56,258)  532/Not reported (n=56,257)   Women: 658/Not reported (n=38,158)  439/Not reported (n=38,159)  384/Not reported (n=38,159)  390/Not reported (n=38,159)  330/Not reported (n=38,158) | Men: 1.00 (ref) 0.95 (0.87-1.04) 0.95 (0.86-1.04) 0.93 (0.84-1.04) 0.93 (0.83-1.04)  Women: 1.00 (ref) 0.94 (0.83-1.07) 0.95 (0.83-1.08) 1.06 (0.93-1.21) 0.98 (0.85-1.13) | Combined, Men/Women |
| Holick et al., 2002* | M | Cohort  (ATBC) | FFQ | Lung (incidence) | Vegetables and fruit | Quintile 1 Quintile 2 Quintile 3 Quintile 4 Quintile 5 | 407/Not reported  362/Not reported  326/Not reported  293/Not reported  256/Not reported | 1.00 (ref) 0.91 (0.79, 1.05) 0.95 (0.82, 1.10) 0.82 (0.71, 0.96) 0.73 (0.62, 0.86) | Combined, Men/Women |
| Gnagnarella et al., 2013 | Both | Cohort  (COSMOS) | FFQ | Lung (incidence) | Vegetables and fruit | Quartile 1 Quartile 2 Quartile 3 Quartile 4 | 58/6,198 (n=1,084)  46/6,234 (n=1,084) 41/6,223 (n=1,084) 33/6,209 (n=1,084) | 1.00 (ref) 0.85 (0.56–1.28) 0.89 (0.58–1.37) 0.85 (0.52–1.36) |  |
| Buchner et al., 2010 | M. W | Cohort  (EPIC) | FFQ | Lung (incidence) | Vegetables and fresh fruits | per 100 g/day | 1,830 (M: 964, W: 866)/ 478,535 (M: 142,649, W: 335,886) | Total: 0.98 (0.96–1.00)  Men: 0.99 (0.96–1.02)  Women: 0.97 (0.93–1.01) | Combined, Men/Women |
| Galeone et al., 2007* | Both | Case-contro (Multi-center hospital-based) | FFQ | Lung (incidence) | Total vegetables and fruit | per 4.1 g/day | 218 (M: 167, W: 51)/ 654 (M: 501, W: 153) | 0.68 (0.57–0.82) | Combined, Men/Women |
| Wakai et al., 2015 | M/W | Cohort  (JPHC I , JPHC II,JACC, MIYAGI) | FFQ | Lung (mortality) | Vegetables and fruit | Quintile 1 Quintile 2 Quintile 3 Quintile 4 Quintile 5 | Men: 298/Not reported 258/Not reported  263/Not reported  289/Not reported 277/Not reported   Women: 91/Not reported 67/Not reported  69/Not reported 91/Not reported 83/Not reported | Men: 1.00 (ref) 0.87 (0.74–1.03) 0.88 (0.75–1.04) 0.97 (0.82–1.14) 0.94 (0.79–1.11)  Women: 1.00 (ref) 0.81 (0.59–1.11) 0.83 (0.60–1.14) 1.10 (0.82–1.49) 0.94 (0.69–1.27) | Combined, Men/Women |
| Galanis et al., 1998* | M/W | Cohort (Hawaii-Japan DOH Survey) | FFQ | Stomach (incidence) | Raw vegetables and fresh fruit | 0-7 times/week 8-13 times/week 14+ times/week | Men: 18/Not reported (n=1,156) 10/Not reported (n=1,742) 36/Not reported (n=2,712)  Women: 6/Not reported (n=1,028) 15/Not reported (n=1,907) 23/Not reported (n=3,362) | Total: 0.5 (0.3-0.9)  0.5 (0.3-0.8)  Men: 0.3 (0.1-0.6)  0.4 (0.2-0.8)  Women: 1.2 (0.4-3.0)  0.7 (0.3-1.7) | Combined, Men/Women |
| Freedman et al., 2008 | M/W | Cohort  (NIH-AARP) | FFQ | Stomach (incidence) | Vegetables and fruit | per serving/1000 kcal/day | 394/490,802 | Total: 1.01 (0.95–1.08)  Men: 1.00 (0.93–1.08)  Women: 1.03 (0.92–1.16) | Combined, Men/Women |
| Larsson et al., 2006 | Both | Cohort (SMC, COSM) | FFQ | Stomach (incidence) | Vegetables and fruit | <2.0 servings/d 2.0-3.4 servings/d 3.5-4.9servings/d ≥5.0 servings/d | 33/82,030 34/151,913  33/147,193 39/210,420 | 1.00 (ref) 0.63 (0.38-1.02)  0.66 (0.39-1.10)  0.54 (0.32-0.91) | Combined |
| Gonzalez et al., 2012 | Both | Cohort (EPIC) | Country/centre specific dietary questionnaires | Stomach (incidence) | Vegetables and fruit | per 100 g/day | 683/477,312 | 0.98 (0.94–1.02) | Combined |
| Botterweck et al., 1998 | Both | Cohort (NLCS) | FFQ | Stomach (incidence) | Total vegetables and fruit | Quintile 1 Quintile 2 Quintile 3 Quintile 4 Quintile 5 | 75/3,503 51/3,567 47/3,577  51/3,599 40/3,620 | 1.00 (ref) 0.74 (0.50-1.09)  0.69 (0.47-1.03)  0.81 (0.55-1.20)  0.72 (0.48-1.10) | Combined |
| Pakseresht et al., 2011* | Both | Case-control  (Population-based) | FFQ | Stomach (incidence) | Vegetables and fruit | per 100 g/day | 286 (M: 210, W: 76)/ 590 (M: 427, W: 163) | 0.72 (0.65–0.80) | Combined |
| Jasen et al., 1999 | M | Cohort study  (SCS) | Dietary records | Stomach (mortality) | Vegetables and fruit | per 31.3 g/day | 267/12,763 | 0.90 (0.82-1.24) | Combined, Men/Women |

sRR, Relative risk; CI, Confidence interval

**[Red meat/Colorectal cancer reference list]**

1. Islam Z, Akter S, Kashino I, Mizoue T, Sawada N, Mori N, et al. Meat subtypes and colorectal cancer risk: A pooled analysis of 6 cohort studies in Japan. Cancer science. 2019;110(11):3603-14.

2. Singh PN, Fraser GE. Dietary risk factors for colon cancer in a low-risk population. American journal of epidemiology. 1998;148(8):761-74.

3. Bernstein AM, Song M, Zhang X, Pan A, Wang M, Fuchs CS, et al. Processed and Unprocessed Red Meat and Risk of Colorectal Cancer: Analysis by Tumor Location and Modification by Time. PloS one. 2015;10(8):e0135959.

4. Jones RR, DellaValle CT, Weyer PJ, Robien K, Cantor KP, Krasner S, et al. Ingested nitrate, disinfection by-products, and risk of colon and rectal cancers in the Iowa Women's Health Study cohort. Environment international. 2019;126:242-51.

5. Mehta SS, Arroyave WD, Lunn RM, Park YMM, Boyd WA, Sandler DP. A prospective analysis of red and processed meat consumption and risk of colorectal cancer in women. Cancer epidemiology, biomarkers & prevention : a publication of the American Association for Cancer Research, cosponsored by the American Society of Preventive Oncology. 2019.

6. Pietinen P, Malila N, Virtanen M, Hartman TJ, Tangrea JA, Albanes D, et al. Diet and risk of colorectal cancer in a cohort of Finnish men. Cancer causes & control : CCC. 1999;10(5):387-96.

7. Jarvinen R, Knekt P, Hakulinen T, Rissanen H, Heliovaara M. Dietary fat, cholesterol and colorectal cancer in a prospective study. British journal of cancer. 2001;85(3):357-61.

8. Gilsing AM, Schouten LJ, Goldbohm RA, Dagnelie PC, van den Brandt PA, Weijenberg MP. Vegetarianism, low meat consumption and the risk of colorectal cancer in a population based cohort study. Scientific reports. 2015;5:13484.

9. Larsson SC, Rafter J, Holmberg L, Bergkvist L, Wolk A. Red meat consumption and risk of cancers of the proximal colon, distal colon and rectum: the Swedish Mammography Cohort. International journal of cancer. 2005;113(5):829-34.

10. Diallo A, Deschasaux M, Latino-Martel P, Hercberg S, Galan P, Fassier P, et al. Red and processed meat intake and cancer risk: Results from the prospective NutriNet-Sante cohort study. International journal of cancer. 2018;142(2):230-7.

11. Knüppel A, Papier K, Fensom G, Appleby P, Schmidt JA, Tong T, et al. Meat intake and cancer risk: prospective analyses in UK Biobank2019.

12. Norat T, Bingham S, Ferrari P, Slimani N, Jenab M, Mazuir M, et al. Meat, fish, and colorectal cancer risk: the European Prospective Investigation into cancer and nutrition. Journal of the National Cancer Institute. 2005;97(12):906-16.

13. English DR, MacInnis RJ, Hodge AM, Hopper JL, Haydon AM, Giles GG. Red meat, chicken, and fish consumption and risk of colorectal cancer. Cancer epidemiology, biomarkers & prevention : a publication of the American Association for Cancer Research, cosponsored by the American Society of Preventive Oncology. 2004;13(9):1509-14.

14. Tiemersma EW, Kampman E, Bueno de Mesquita HB, Bunschoten A, van Schothorst EM, Kok FJ, et al. Meat consumption, cigarette smoking, and genetic susceptibility in the etiology of colorectal cancer: results from a Dutch prospective study. Cancer causes & control : CCC. 2002;13(4):383-93.

15. Kim NH, Seol JE, Kim J, Lee BH, Hwang DY, Jeong J, et al. Red meat intake, CYP2E1 and PPARγ polymorphisms, and colorectal cancer risk. European Journal of Cancer Prevention. 2019;28(4):304-10.

16. Kimura Y, Kono S, Toyomura K, Nagano J, Mizoue T, Moore MA, et al. Meat, fish and fat intake in relation to subsite-specific risk of colorectal cancer: The Fukuoka Colorectal Cancer Study. Cancer science. 2007;98(4):590-7.

17. Saliba W, Rennert HS, Gronich N, Gruber SB, Rennert G. Red meat and processed meat intake and risk of colorectal cancer: A population-based case-control study. European Journal of Cancer Prevention. 2019;28(4):287-93.

18. Wei EK, Giovannucci E, Wu K, Rosner B, Fuchs CS, Willett WC, et al. Comparison of risk factors for colon and rectal cancer. International journal of cancer. 2004;108(3):433-42.

19. Vulcan A, Manjer J, Ericson U, Ohlsson B. Intake of different types of red meat, poultry, and fish and incident colorectal cancer in women and men: Results from the Malmö diet and cancer study. Food and Nutrition Research. 2017;61.

20. Parr CL, Hjartaker A, Lund E, Veierod MB. Meat intake, cooking methods and risk of proximal colon, distal colon and rectal cancer: the Norwegian Women and Cancer (NOWAC) cohort study. International journal of cancer. 2013;133(5):1153-63.

**[Processed meat/Colorectal cancer reference list]**

1. Islam Z, Akter S, Kashino I, Mizoue T, Sawada N, Mori N, et al. Meat subtypes and colorectal cancer risk: A pooled analysis of 6 cohort studies in Japan. Cancer science. 2019;110(11):3603-14.

2. Lin J, Zhang SM, Cook NR, Lee IM, Buring JE. Dietary fat and fatty acids and risk of colorectal cancer in women. American journal of epidemiology. 2004;160(10):1011-22.

3. Chao A, Thun MJ, Connell CJ, McCullough ML, Jacobs EJ, Flanders WD, et al. Meat consumption and risk of colorectal cancer. Jama. 2005;293(2):172-82.

4. Ollberding NJ, Wilkens LR, Henderson BE, Kolonel LN, Le Marchand L. Meat consumption, heterocyclic amines and colorectal cancer risk: the Multiethnic Cohort Study. International journal of cancer. 2012;131(7):E1125-33.

5. Jones RR, DellaValle CT, Weyer PJ, Robien K, Cantor KP, Krasner S, et al. Ingested nitrate, disinfection by-products, and risk of colon and rectal cancers in the Iowa Women's Health Study cohort. Environment international. 2019;126:242-51.

6. Flood A, Velie EM, Sinha R, Chaterjee N, Lacey Jr JV, Schairer C, et al. Meat, fat, and their subtypes as risk factors for colorectal cancer in a prospective cohort of women. American journal of epidemiology. 2003;158(1):59-68.

7. Bernstein AM, Song M, Zhang X, Pan A, Wang M, Fuchs CS, et al. Processed and Unprocessed Red Meat and Risk of Colorectal Cancer: Analysis by Tumor Location and Modification by Time. PloS one. 2015;10(8):e0135959.

8. Etemadi A, Abnet CC, Graubard BI, Beane-Freeman L, Freedman ND, Liao L, et al. Anatomical subsite can modify the association between meat and meat compounds and risk of colorectal adenocarcinoma: Findings from three large US cohorts. International journal of cancer. 2018;143(9):2261-70.

9. Mehta SS, Arroyave WD, Lunn RM, Park YMM, Boyd WA, Sandler DP. A prospective analysis of red and processed meat consumption and risk of colorectal cancer in women. Cancer epidemiology, biomarkers & prevention : a publication of the American Association for Cancer Research, cosponsored by the American Society of Preventive Oncology. 2019.

10. Gaard M, Tretli S, Loken EB. Dietary factors and risk of colon cancer: a prospective study of 50,535 young Norwegian men and women. European journal of cancer prevention : the official journal of the European Cancer Prevention Organisation (ECP). 1996;5(6):445-54.

11. Pietinen P, Malila N, Virtanen M, Hartman TJ, Tangrea JA, Albanes D, et al. Diet and risk of colorectal cancer in a cohort of Finnish men. Cancer causes & control : CCC. 1999;10(5):387-96.

12. English DR, MacInnis RJ, Hodge AM, Hopper JL, Haydon AM, Giles GG. Red meat, chicken, and fish consumption and risk of colorectal cancer. Cancer epidemiology, biomarkers & prevention : a publication of the American Association for Cancer Research, cosponsored by the American Society of Preventive Oncology. 2004;13(9):1509-14.

13. Larsson SC, Rafter J, Holmberg L, Bergkvist L, Wolk A. Red meat consumption and risk of cancers of the proximal colon, distal colon and rectum: the Swedish Mammography Cohort. International journal of cancer. 2005;113(5):829-34.

14. Egeberg R, Olsen A, Christensen J, Halkjaer J, Jakobsen MU, Overvad K, et al. Associations between red meat and risks for colon and rectal cancer depend on the type of red meat consumed. The Journal of nutrition. 2013;143(4):464-72.

15. Parr CL, Hjartaker A, Lund E, Veierod MB. Meat intake, cooking methods and risk of proximal colon, distal colon and rectal cancer: the Norwegian Women and Cancer (NOWAC) cohort study. International journal of cancer. 2013;133(5):1153-63.

16. Vulcan A, Manjer J, Ericson U, Ohlsson B. Intake of different types of red meat, poultry, and fish and incident colorectal cancer in women and men: Results from the Malmö diet and cancer study. Food and Nutrition Research. 2017;61.

17. Diallo A, Deschasaux M, Latino-Martel P, Hercberg S, Galan P, Fassier P, et al. Red and processed meat intake and cancer risk: Results from the prospective NutriNet-Santé cohort study. International journal of cancer. 2018;142(2):230-7.

18. Bradbury KE, Murphy N, Key TJ. Diet and colorectal cancer in UK Biobank: a prospective study. International journal of epidemiology. 2019.

19. Balder HF, De Vogel J, Jansen MCJF, Weijenberg MP, Van Den Brandt PA, Westenbrink S, et al. Heme and chlorophyll intake and risk of colorectal cancer in the Netherlands cohort study. Cancer Epidemiology Biomarkers and Prevention. 2006;15(4):717-25.

20. Spencer EA, Key TJ, Appleby PN, Dahm CC, Keogh RH, Fentiman IS, et al. Meat, poultry and fish and risk of colorectal cancer: pooled analysis of data from the UK dietary cohort consortium. Cancer causes & control : CCC. 2010;21(9):1417-25.

21. Kimura Y, Kono S, Toyomura K, Nagano J, Mizoue T, Moore MA, et al. Meat, fish and fat intake in relation to subsite-specific risk of colorectal cancer: The Fukuoka Colorectal Cancer Study. Cancer science. 2007;98(4):590-7.

22. Saliba W, Rennert HS, Gronich N, Gruber SB, Rennert G. Red meat and processed meat intake and risk of colorectal cancer: a population-based case-control study. European journal of cancer prevention : the official journal of the European Cancer Prevention Organisation (ECP). 2019;28(4):287-93.

23. Kojima M, Wakai K, Tamakoshi K, Tokudome S, Toyoshima H, Watanabe Y, et al. Diet and colorectal cancer mortality: results from the Japan Collaborative Cohort Study. Nutrition and cancer. 2004;50(1):23-32.

24. Wei EK, Giovannucci E, Wu K, Rosner B, Fuchs CS, Willett WC, et al. Comparison of risk factors for colon and rectal cancer. International journal of cancer. 2004;108(3):433-42.

**[Dietary fiber/Colorectal cancer reference list]**

1. Kunzmann AT, Coleman HG, Huang WY, Kitahara CM, Cantwell MM, Berndt SI. Dietary fiber intake and risk of colorectal cancer and incident and recurrent adenoma in the Prostate, Lung, Colorectal, and Ovarian Cancer Screening Trial. Am J Clin Nutr. 2015;102(4):881-90.

2. Navarro SL, Neuhouser ML, Cheng TD, Tinker LF, Shikany JM, Snetselaar L, et al. The Interaction between Dietary Fiber and Fat and Risk of Colorectal Cancer in the Women's Health Initiative. Nutrients. 2016;8(12).

3. Otani T, Iwasaki M, Ishihara J, Sasazuki S, Inoue M, Tsugane S. Dietary fiber intake and subsequent risk of colorectal cancer: the Japan Public Health Center-based prospective study. Int J Cancer. 2006;119(6):1475-80.

4. Park SY, Wilkens LR, Kolonel LN, Henderson BE, Le Marchand L. Inverse associations of dietary fiber and menopausal hormone therapy with colorectal cancer risk in the Multiethnic Cohort Study. Int J Cancer. 2016;139(6):1241-50.

5. Schatzkin A, Mouw T, Park Y, Subar AF, Kipnis V, Hollenbeck A, et al. Dietary fiber and whole-grain consumption in relation to colorectal cancer in the NIH-AARP Diet and Health Study. Am J Clin Nutr. 2007;85(5):1353-60.

6. Shin A, Li H, Shu XO, Yang G, Gao YT, Zheng W. Dietary intake of calcium, fiber and other micronutrients in relation to colorectal cancer risk: Results from the Shanghai Women's Health Study. International Journal of Cancer. 2006;119(12):2938-42.

7. Wakai K, Date C, Fukui M, Tamakoshi K, Watanabe Y, Hayakawa N, et al. Dietary fiber and risk of colorectal cancer in the Japan collaborative cohort study. Cancer Epidemiol Biomarkers Prev. 2007;16(4):668-75.

8. Gaard M, Tretli S, Løken E. Dietary factors and risk of colon cancer: a prospective study of 50,535 young Norwegian men and women. European journal of cancer prevention: the official journal of the European Cancer Prevention Organisation (ECP). 1996;5(6):445-54.

9. Andersen V, Halekoh U, Tjonneland A, Vogel U, Kopp TI. Intake of Red and Processed Meat, Use of Non-Steroid Anti-Inflammatory Drugs, Genetic Variants and Risk of Colorectal Cancer: A Prospective Study of the Danish "Diet, Cancer and Health" Cohort. Int J Mol Sci. 2019;20(5).

10. Bradbury KE, Murphy N, Key TJ. Diet and colorectal cancer in UK Biobank: a prospective study. Int J Epidemiol. 2019.

11. Murphy N, Norat T, Ferrari P, Jenab M, Bueno-de-Mesquita B, Skeie G, et al. Dietary fibre intake and risks of cancers of the colon and rectum in the European prospective investigation into cancer and nutrition (EPIC). PLoS ONE. 2012;7(6).

12. Sanjoaquin MA, Appleby PN, Thorogood M, Mann JI, Key TJ. Nutrition, lifestyle and colorectal cancer incidence: a prospective investigation of 10998 vegetarians and non-vegetarians in the United Kingdom. Br J Cancer. 2004;90(1):118-21.

13. Uchida K, Kono S, Yin G, Toyomura K, Nagano J, Mizoue T, et al. Dietary fiber, source foods and colorectal cancer risk: the Fukuoka Colorectal Cancer Study. Scand J Gastroenterol. 2010;45(10):1223-31.

14. Jansen MC, Bueno-de-Mesquita HB, Buzina R, Fidanza F, Menotti A, Blackburn H, et al. Dietary fiber and plant foods in relation to colorectal cancer mortality: the Seven Countries Study. Int J Cancer. 1999;81(2):174-9.

15. Song M, Wu K, Meyerhardt JA, Ogino S, Wang M, Fuchs CS, et al. Fiber Intake and Survival After Colorectal Cancer Diagnosis. JAMA Oncol. 2018;4(1):71-9.

16. McCullough ML, Robertson AS, Chao A, Jacobs EJ, Stampfer MJ, Jacobs DR, et al. A prospective study of whole grains, fruits, vegetables and colon cancer risk. Cancer Causes Control. 2003;14(10):959-70.

17. He X, Wu K, Zhang X, Nishihara R, Cao Y, Fuchs CS, et al. Dietary intake of fiber, whole grains and risk of colorectal cancer: An updated analysis according to food sources, tumor location and molecular subtypes in two large US cohorts. International Journal of Cancer. 2019;145(11):3040-51.

18. Lin J, Zhang SM, Cook NR, Rexrode KM, Liu S, Manson JE, et al. Dietary intakes of fruit, vegetables, and fiber, and risk of colorectal cancer in a prospective cohort of women (United States). Cancer causes & control. 2005;16(3):225-33.

19. Mai V, Flood A, Peters U, Lacey Jr JV, Schairer C, Schatzkin A. Dietary fibre and risk of colorectal cancer in the Breast Cancer Detection Demonstration Project (BCDDP) follow-up cohort. International Journal of Epidemiology. 2003;32(2):234-9.

20. McCarl M, Harnack L, Limburg PJ, Anderson KE, Folsom AR. Incidence of colorectal cancer in relation to glycemic index and load in a cohort of women. Cancer Epidemiology and Prevention Biomarkers. 2006;15(5):892-6.

21. Pietinen P, Malila N, Virtanen M, Hartman TJ, Tangrea JA, Albanes D, et al. Diet and risk of colorectal cancer in a cohort of Finnish men. Cancer Causes Control. 1999;10(5):387-96.

22. Terry P, Giovannucci E, Michels KB, Bergkvist L, Hansen H, Holmberg L, et al. Fruit, vegetables, dietary fiber, and risk of colorectal cancer. J Natl Cancer Inst. 2001;93(7):525-33.

23. Huang J, Fang YJ, Xu M, Luo H, Zhang NQ, Huang WQ, et al. Carbohydrate, dietary glycaemic index and glycaemic load, and colorectal cancer risk: A case-control study in China. British Journal of Nutrition. 2018;119(8):937-48.

**[Salted vegetable/Gastric cancer reference list]**

1. Cai L, Zheng ZL, Zhang ZF. Risk factors for the gastric cardia cancer: a case-control study in Fujian Province. World journal of gastroenterology. 2003;9(2):214-8.

2. Galanis DJ, Kolonel LN, Lee J, Nomura A. Intakes of selected foods and beverages and the incidence of gastric cancer among the Japanese residents of Hawaii: a prospective study. International journal of epidemiology. 1998;27(2):173-80.

3. Hamada GS, Kowalski LP, Nishimoto IN, Rodrigues JJ, Iriya K, Sasazuki S, et al. Risk factors for stomach cancer in Brazil (II): a case-control study among Japanese Brazilians in São Paulo. Japanese journal of clinical oncology. 2002;32(8):284-90.

4. Iso H, Kubota Y. Nutrition and disease in the Japan Collaborative Cohort Study for Evaluation of Cancer (JACC). Asian Pacific journal of cancer prevention : APJCP. 2007;8 Suppl:35-80.

5. Kato I, Tominaga S, Ito Y, Kobayashi S, Yoshii Y, Matsuura A, et al. A prospective study of atrophic gastritis and stomach cancer risk. Japanese journal of cancer research : Gann. 1992;83(11):1137-42.

6. Kato I, Tominaga S, Matsumoto K. A prospective study of stomach cancer among a rural Japanese population: a 6-year survey. Japanese journal of cancer research : Gann. 1992;83(6):568-75.

7. Machida-Montani A, Sasazuki S, Inoue M, Natsukawa S, Shaura K, Koizumi Y, et al. Association of Helicobacter pylori infection and environmental factors in non-cardia gastric cancer in Japan. Gastric cancer : official journal of the International Gastric Cancer Association and the Japanese Gastric Cancer Association. 2004;7(1):46-53.

8. Ngoan LT, Mizoue T, Fujino Y, Tokui N, Yoshimura T. Dietary factors and stomach cancer mortality. British journal of cancer. 2002;87(1):37-42.

9. Nomura A, Grove JS, Stemmermann GN, Severson RK. A prospective study of stomach cancer and its relation to diet, cigarettes, and alcohol consumption. Cancer Res. 1990;50(3):627-31.

10. Sauvaget C, Lagarde F, Nagano J, Soda M, Koyama K, Kodama K. Lifestyle factors, radiation and gastric cancer in atomic-bomb survivors (Japan). Cancer causes & control : CCC. 2005;16(7):773-80.

11. Sun CQ, Chang YB, Cui LL, Chen JJ, Sun N, Zhang WJ, et al. A population-based case-control study on risk factors for gastric cardia cancer in rural areas of Linzhou. Asian Pacific journal of cancer prevention : APJCP. 2013;14(5):2897-901.

**[Salted fish/Gastric cancer reference list]**

1. Cai L, Zheng ZL, Zhang ZF. Risk factors for the gastric cardia cancer: a case-control study in Fujian Province. World journal of gastroenterology. 2003;9(2):214-8.

2. Iso H, Kubota Y. Nutrition and disease in the Japan Collaborative Cohort Study for Evaluation of Cancer (JACC). Asian Pacific journal of cancer prevention : APJCP. 2007;8 Suppl:35-80.

3. Ngoan LT, Mizoue T, Fujino Y, Tokui N, Yoshimura T. Dietary factors and stomach cancer mortality. British journal of cancer. 2002;87(1):37-42.

4. Pakseresht M, Forman D, Malekzadeh R, Yazdanbod A, West RM, Greenwood DC, et al. Dietary habits and gastric cancer risk in north-west Iran. Cancer causes & control : CCC. 2011;22(5):725-36.

5. Takachi R, Inoue M, Shimazu T, Sasazuki S, Ishihara J, Sawada N, et al. Consumption of sodium and salted foods in relation to cancer and cardiovascular disease: the Japan Public Health Center-based Prospective Study. The American journal of clinical nutrition. 2010;91(2):456-64.

**[Salted fish/Nasopharyngeal cancer reference list]**

1. Yuan JM, Wang XL, Xiang YB, Gao YT, Ross RK, Yu MC. Preserved foods in relation to risk of nasopharyngeal carcinoma in Shanghai, China. International journal of cancer. 2000;85(3):358-63.

2. Ren ZF, Liu WS, Qin HD, Xu YF, Yu DD, Feng QS, et al. Effect of family history of cancers and environmental factors on risk of nasopharyngeal carcinoma in Guangdong, China. Cancer epidemiology. 2010;34(4):419-24.

3. Barrett D, Ploner A, Chang ET, Liu Z, Zhang CX, Liu Q, et al. Past and recent salted fish and preserved food intakes are weakly associated with nasopharyngeal carcinoma risk in adults in southern China. Journal of Nutrition. 2019;149(9):1596-605.

4. Sriamporn S, Vatanasapt V, Pisani P, Yongchaiyudha S, Rungpitarangsri V. Environmental risk factors for nasopharyngeal carcinoma: a case-control study in northeastern Thailand. Cancer epidemiology, biomarkers & prevention : a publication of the American Association for Cancer Research, cosponsored by the American Society of Preventive Oncology. 1992;1(5):345-8.

5. Yang XR, Diehl S, Pfeiffer R, Chen CJ, Hsu WL, Dosemeci M, et al. Evaluation of risk factors for nasopharyngeal carcinoma in high-risk nasopharyngeal carcinoma families in Taiwan. Cancer epidemiology, biomarkers & prevention : a publication of the American Association for Cancer Research, cosponsored by the American Society of Preventive Oncology. 2005;14(4):900-5.

6. Yong SK, Ha TC, Yeo MC, Gaborieau V, McKay JD, Wee J. Associations of lifestyle and diet with the risk of nasopharyngeal carcinoma in Singapore: a case-control study. Chin J Cancer. 2017;36(1):3.

7. Xu FH, Xiong D, Xu YF, Cao SM, Xue WQ, Qin HD, et al. An epidemiological and molecular study of the relationship between smoking, risk of nasopharyngeal carcinoma, and Epstein-Barr virus activation. J Natl Cancer Inst. 2012;104(18):1396-410.

**[Non-starch vegetable/Aerodigestive and some other (colorectal, gastric, lung) cancer reference list]**

1. Wie GA, Cho YA, Kang HH, Ryu KA, Yoo MK, Kim YA, et al. Red meat consumption is associated with an increased overall cancer risk: a prospective cohort study in Korea. The British journal of nutrition. 2014;112(2):238-47.

2. Yamaji T, Inoue M, Sasazuki S, Iwasaki M, Kurahashi N, Shimazu T, et al. Fruit and vegetable consumption and squamous cell carcinoma of the esophagus in Japan: the JPHC study. International journal of cancer. 2008;123(8):1935-40.

3. Wakai K, Sugawara Y, Tsuji I, Tamakoshi A, Shimazu T, Matsuo K, et al. Risk of lung cancer and consumption of vegetables and fruit in Japanese: A pooled analysis of cohort studies in Japan. Cancer science. 2015;106(8):1057-65.

4. Vogtmann E, Xiang YB, Li HL, Levitan EB, Yang G, Waterbor JW, et al. Fruit and vegetable intake and the risk of colorectal cancer: results from the Shanghai Men's Health Study. Cancer causes & control : CCC. 2013;24(11):1935-45.

5. Lin J, Zhang SM, Cook NR, Rexrode KM, Liu S, Manson JE, et al. Dietary intakes of fruit, vegetables, and fiber, and risk of colorectal cancer in a prospective cohort of women (United States). Cancer causes & control : CCC. 2005;16(3):225-33.

6. McCarl M, Harnack L, Limburg PJ, Anderson KE, Folsom AR. Incidence of colorectal cancer in relation to glycemic index and load in a cohort of women. Cancer epidemiology, biomarkers & prevention : a publication of the American Association for Cancer Research, cosponsored by the American Society of Preventive Oncology. 2006;15(5):892-6.

7. Park Y, Subar AF, Kipnis V, Thompson FE, Mouw T, Hollenbeck A, et al. Fruit and vegetable intakes and risk of colorectal cancer in the NIH-AARP diet and health study. American journal of epidemiology. 2007;166(2):170-80.

8. Freedman ND, Subar AF, Hollenbeck AR, Leitzmann MF, Schatzkin A, Abnet CC. Fruit and vegetable intake and gastric cancer risk in a large United States prospective cohort study. Cancer causes & control : CCC. 2008;19(5):459-67.

9. Nomura AM, Wilkens LR, Murphy SP, Hankin JH, Henderson BE, Pike MC, et al. Association of vegetable, fruit, and grain intakes with colorectal cancer: the Multiethnic Cohort Study. The American journal of clinical nutrition. 2008;88(3):730-7.

10. Slatore CG, Littman AJ, Au DH, Satia JA, White E. Long-term use of supplemental multivitamins, vitamin C, vitamin E, and folate does not reduce the risk of lung cancer. American journal of respiratory and critical care medicine. 2008;177(5):524-30.

11. Wright ME, Park Y, Subar AF, Freedman ND, Albanes D, Hollenbeck A, et al. Intakes of fruit, vegetables, and specific botanical groups in relation to lung cancer risk in the NIH-AARP Diet and Health Study. American journal of epidemiology. 2008;168(9):1024-34.

12. Makarem N, Lin Y, Bandera EV, Jacques PF, Parekh N. Concordance with World Cancer Research Fund/American Institute for Cancer Research (WCRF/AICR) guidelines for cancer prevention and obesity-related cancer risk in the Framingham Offspring cohort (1991-2008). Cancer causes & control : CCC. 2015;26(2):277-86.

13. Kunzmann AT, Coleman HG, Huang WY, Cantwell MM, Kitahara CM, Berndt SI. Fruit and vegetable intakes and risk of colorectal cancer and incident and recurrent adenomas in the PLCO cancer screening trial. International journal of cancer. 2016;138(8):1851-61.

14. Botterweck AA, van den Brandt PA, Goldbohm RA. A prospective cohort study on vegetable and fruit consumption and stomach cancer risk in The Netherlands. American journal of epidemiology. 1998;148(9):842-53.

15. Voorrips LE, Goldbohm RA, van Poppel G, Sturmans F, Hermus RJ, van den Brandt PA. Vegetable and fruit consumption and risks of colon and rectal cancer in a prospective cohort study: The Netherlands Cohort Study on Diet and Cancer. American journal of epidemiology. 2000;152(11):1081-92.

16. Larsson SC, Bergkvist L, Wolk A. Fruit and vegetable consumption and incidence of gastric cancer: a prospective study. Cancer epidemiology, biomarkers & prevention : a publication of the American Association for Cancer Research, cosponsored by the American Society of Preventive Oncology. 2006;15(10):1998-2001.

17. Gnagnarella P, Maisonneuve P, Bellomi M, Rampinelli C, Bertolotti R, Spaggiari L, et al. Red meat, Mediterranean diet and lung cancer risk among heavy smokers in the COSMOS screening study. Annals of oncology : official journal of the European Society for Medical Oncology. 2013;24(10):2606-11.

18. Maasland DH, van den Brandt PA, Kremer B, Goldbohm RA, Schouten LJ. Consumption of vegetables and fruits and risk of subtypes of head-neck cancer in the Netherlands Cohort Study. International journal of cancer. 2015;136(5):E396-409.

19. Boeing H, Dietrich T, Hoffmann K, Pischon T, Ferrari P, Lahmann PH, et al. Intake of fruits and vegetables and risk of cancer of the upper aero-digestive tract: the prospective EPIC-study. Cancer causes & control : CCC. 2006;17(7):957-69.

20. van Duijnhoven FJ, Bueno-De-Mesquita HB, Ferrari P, Jenab M, Boshuizen HC, Ros MM, et al. Fruit, vegetables, and colorectal cancer risk: the European Prospective Investigation into Cancer and Nutrition. The American journal of clinical nutrition. 2009;89(5):1441-52.

21. Buchner FL, Bueno-de-Mesquita HB, Linseisen J, Boshuizen HC, Kiemeney LA, Ros MM, et al. Fruits and vegetables consumption and the risk of histological subtypes of lung cancer in the European Prospective Investigation into Cancer and Nutrition (EPIC). Cancer causes & control : CCC. 2010;21(3):357-71.

22. Gonzalez CA, Lujan-Barroso L, Bueno-de-Mesquita HB, Jenab M, Duell EJ, Agudo A, et al. Fruit and vegetable intake and the risk of gastric adenocarcinoma: a reanalysis of the European Prospective Investigation into Cancer and Nutrition (EPIC-EURGAST) study after a longer follow-up. International journal of cancer. 2012;131(12):2910-9.

23. Lee SA, Shu XO, Yang G, Li H, Gao YT, Zheng W. Animal origin foods and colorectal cancer risk: a report from the Shanghai Women's Health Study. Nutrition and cancer. 2009;61(2):194-205.

24. Jansen MC, Bueno-de-Mesquita HB, Rasanen L, Fidanza F, Menotti A, Nissinen A, et al. Consumption of plant foods and stomach cancer mortality in the seven countries study. Is grain consumption a risk factor? Seven Countries Study Research Group. Nutrition and cancer. 1999;34(1):49-55.

25. Galanis DJ, Kolonel LN, Lee J, Nomura A. Intakes of selected foods and beverages and the incidence of gastric cancer among the Japanese residents of Hawaii: a prospective study. International journal of epidemiology. 1998;27(2):173-80.
